# Soil Mineral Composition Matters: Response of Microbial Communities to Phenanthrene and Plant Litter Addition in Long-Term Matured Artificial Soils

**DOI:** 10.1371/journal.pone.0106865

**Published:** 2014-09-15

**Authors:** Doreen Babin, Cordula Vogel, Sebastian Zühlke, Michael Schloter, Geertje Johanna Pronk, Katja Heister, Michael Spiteller, Ingrid Kögel-Knabner, Kornelia Smalla

**Affiliations:** 1 Institute for Epidemiology and Pathogen Diagnostics, Julius Kühn-Institut - Federal Research Centre for Cultivated Plants (JKI), Braunschweig, Germany; 2 Lehrstuhl für Bodenkunde, Technische Universität München, Freising-Weihenstephan, Germany; 3 Institut für Umweltforschung (INFU), Lehrstuhl für Umweltchemie und Analytische Chemie der Fakultät für Chemie und Chemische Biologie, Technische Universität Dortmund, Dortmund, Germany; 4 Research Unit for Environmental Genomics, Helmholtz Zentrum München, German Research Center for Environmental Health, Neuherberg, Germany; 5 Institute for Advanced Study, Technische Universität München, Garching, Germany; NERC Centre for Ecology & Hydrology, United Kingdom

## Abstract

The fate of polycyclic aromatic hydrocarbons (PAHs) in soil is determined by a suite of biotic and abiotic factors, and disentangling their role in the complex soil interaction network remains challenging. Here, we investigate the influence of soil composition on the microbial community structure and its response to the spiked model PAH compound phenanthrene and plant litter. We used long-term matured artificial soils differing in type of clay mineral (illite, montmorillonite) and presence of charcoal or ferrihydrite. The soils received an identical soil microbial fraction and were incubated for more than two years with two sterile manure additions. The matured artificial soils and a natural soil were subjected to the following spiking treatments: (I) phenanthrene, (II) litter, (III) litter + phenanthrene, (IV) unspiked control. Total community DNA was extracted from soil sampled on the day of spiking, 7, 21, and 63 days after spiking. Bacterial 16S rRNA gene and fungal internal transcribed spacer amplicons were quantified by qPCR and subjected to denaturing gradient gel electrophoresis (DGGE). DGGE analysis revealed that the bacterial community composition, which was strongly shaped by clay minerals after more than two years of incubation, changed in response to spiked phenanthrene and added litter. DGGE and qPCR showed that soil composition significantly influenced the microbial response to spiking. While fungal communities responded only in presence of litter to phenanthrene spiking, the response of the bacterial communities to phenanthrene was less pronounced when litter was present. Interestingly, microbial communities in all artificial soils were more strongly affected by spiking than in the natural soil, which might indicate the importance of higher microbial diversity to compensate perturbations. This study showed the influence of soil composition on the microbiota and their response to phenanthrene and litter, which may increase our understanding of complex interactions in soils for bioremediation applications.

## Introduction

Bioremediation refers to different cleanup strategies using living organisms for the removal of environmental pollutants, such as polycyclic aromatic hydrocarbons (PAHs), from soils contaminated by anthropogenic activities. Different parameters can severely affect the efficiency of the methods, as for instance the exposure time of the contaminant in soil [1], soil structure [2], pH [3], temperature [3], sorptive interfaces [4], and the organic carbon content [5]. In addition, the accessibility to oxygen may be of importance, since the fastest and most often used microbial pathway of PAH degradation involves the oxidation of the ring-structure via dioxygenases [6]. The concentration and availability of nutrients, such as nitrogen and phosphorus, represent another factor influencing the rate of biodegradation in contaminated soils [3], [6]. To enhance the bioremediation efficiency, a current method is the addition of straw, compost, or manure to polluted soils which improves soil structure, oxygen transfer and provides energy sources for the soil microbiota [7].

Apart from those single factors and existing strategies to tackle them, the efficient application of biodegradation in the environment is still challenged by two scientific frontiers as reviewed recently by Jeon & Madsen [8]: On the one hand, although many studies exist on reactions of pollutants with clays and soil organic matter (OM), e.g. [2], [5], [9], [10], [11], we lack a comprehensive understanding of these abiotic soil interaction processes. On the other hand, there is restricted knowledge of the interaction of pollutants with native soil microbial communities within the natural soil system. So far, only a few studies have been carried out to compare the consequences of contamination on the microbiota in different soils. For instance, Bundy et al. [12] and Ding et al. [13] showed previously that each soil type has its individual microbial response to pollutants. In this respect, it is necessary to consider the role of the interplay between microbes and their physical soil environment [14]. This is of particular interest since the different organic, inorganic and biological soil components are in close contact and form complex, so-called biogeochemical interfaces where important ecosystem processes take place [15].

The influence of the soil mineral composition on the response of microbial communities to pollutants and the resulting effect on the biodegradative potential has been poorly addressed so far. It is known that different soil components, such as minerals and charcoal, can influence metabolic activity, the establishment and the microenvironment of the soil microbiota [16], [17], [18], [19]; but might the soil composition interfere also with the microbial response to and biodegradation of pollutants? In order to answer this question, artificial soils are a good tool to simulate natural soil environments [20], [21], [22], [23]. The design of artificial soils with defined compositions makes it possible to disentangle the influence of certain factors (e.g., soil components, soil OM, microbiota, pollutants) on a particular soil process. Based on this, we recently investigated the effect of the soil mineral composition and the presence of charcoal on the early soil interface development. We observed the fast formation of organo-mineral associations [22] and the dynamic effects of charcoal, clay minerals and metal oxides on the microbial community establishment [13], [24]. After spiking one year-incubated artificial soils with the model PAH compound phenanthrene, bacteria from different artificial soils showed similar and soil composition-dependent responses to the pollutant [24]. Recently, Vogel et al. [25] reported on the maturation of artificial soils over a long incubation period of 842 days. The authors observed that clay minerals are the major long-term driver of bacterial communities and that artificial soils developed to soil-like systems [25]. We hypothesized that the establishment of these soil composition-driven microbial communities and interfaces over such a long period causes different responses of the microbiota to spiked phenanthrene. Furthermore, we assumed that plant litter addition stimulates the established microbial communities and their response to phenanthrene.

**Figure 1:**
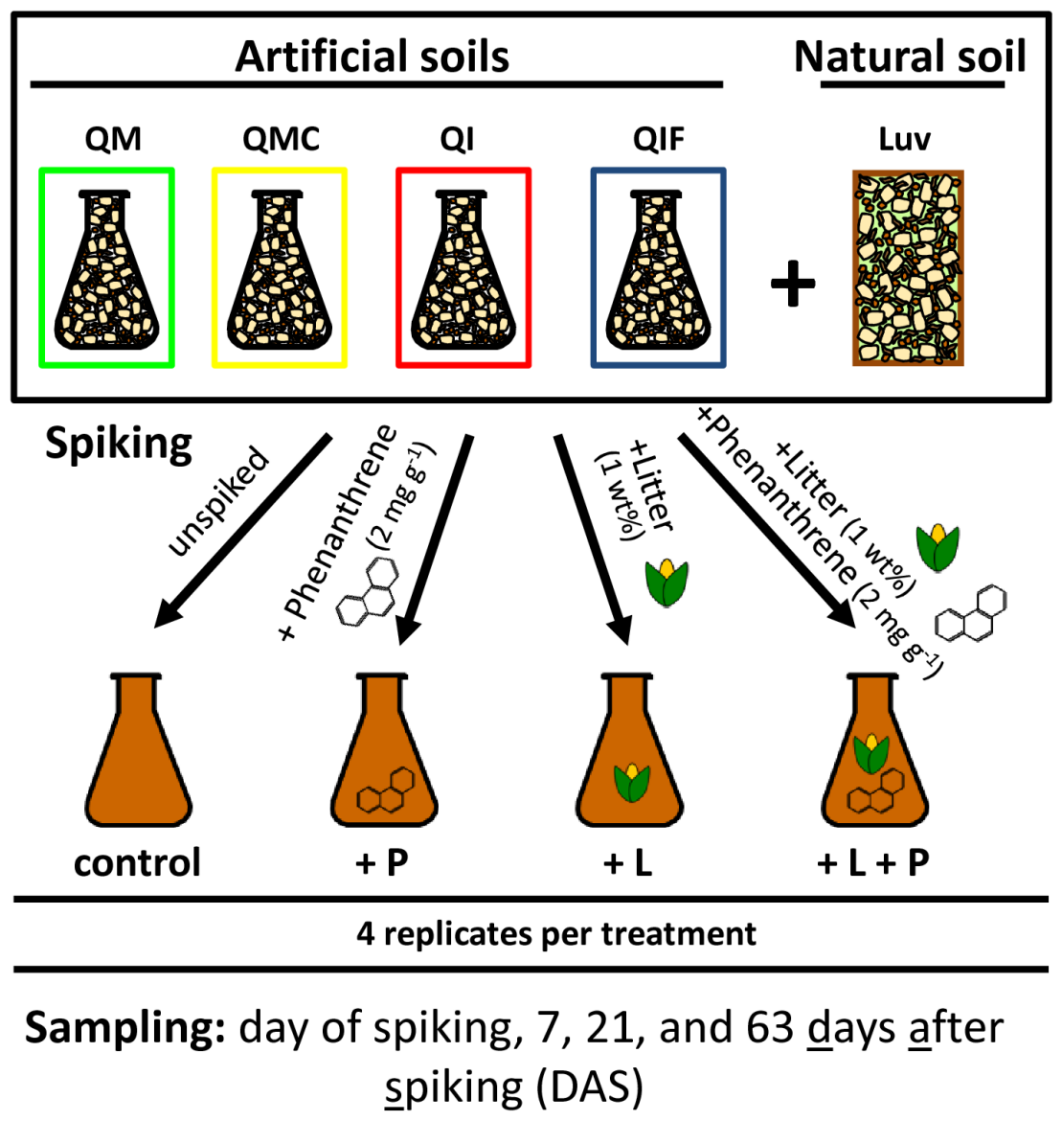
Experimental design. Long-term matured artificial soils QM, QMC, QI, QIF and the natural soil were subjected to following treatments: unspiked control, phenanthrene (+P), plant litter (+L), litter and phenanthrene (+L+P). Each treatment consisted of four replicates. Sampling was carried out on the day of spiking, 7, 21 and 63 days after spiking (DAS). Q-quartz, M-montmorillonite, C-charcoal, I-illite, F-ferrihydrite, Luv-natural soil (Luvisol).

We therefore conducted a spiking experiment on artificial soils incubated for 842 days with four different treatments: phenanthrene (+P), litter (+L), litter and phenanthrene (+L+P), unspiked control (Figure 1). Four soil compositions were used based on the results of the aforementioned studies [19], [24], [25]: two soils differed in the type of clay mineral (montmorillonite [M] or illite [I]), one soil contained charcoal (C) and one soil had the metal oxide ferrihydrite (F) as additional soil component. The microbiota in differently treated artificial soils was compared to a natural soil which was spiked similarly. We sampled and extracted total community DNA (TC-DNA) on the day of spiking, 7 days after spiking (DAS), 21 DAS, and 63 DAS. The responses of the microbial communities to the spiking were studied by denaturing gradient gel electrophoresis (DGGE) fingerprints and quantitative real-time PCR (qPCR) of 16S rRNA gene and internal transcribed spacer (ITS) fragments. This study shows for the first time the effect of spiked phenanthrene and litter amendment on microbial communities which established over a long-term period as a function of the soil mineral composition and presence of charcoal.

## Materials and Methods

### 1. Soils used in this study

Design and model materials of the artificial soil compositions were previously described in detail by Pronk et al. [22] and Vogel et al. [25]. The following four artificial soil compositions were used in this experiment: QM (94% quartz +6% montmorillonite), QI (92% quartz +8% illite), QMC (94% quartz +4% montmorillonite +2% charcoal), and QIF (92% quartz +7% illite +1% ferrihydrite). Sand- and silt-sized quartz was used as structure material similarly for all artificial soil compositions. Sterile manure (4.5 wt%) was used as initial OM input. The inoculant was derived by water extraction from a topsoil of an agricultural site in southern Germany (Scheyern; 48°N, 11°E) which has been classified as a Luvisol. The inoculant was added to the soil compositions after an abiotic conditioning time of three days with 0.01 M CaCl_2_ as artificial soil solution. Soils were incubated under constant environmental conditions (20°C, constant water holding capacity of 60%, in the dark) and gently mixed weekly for homogenization. After 562 days of incubation, the addition of sterile manure (4.5 wt%) was repeated to provide a fresh OM source, since previous experiments suggested the decrease of the microbial activity and macro-aggregation due to limited nutrients [22]. Soils were incubated up to 842 days and a detailed characterization of soil parameters as well as of microbial communities established during that incubation time can be found in Vogel et al. [25]. The natural soil used in this study was an arable topsoil (Ap) from a Luvisol (Luv) and was collected from the same site as the soil used for the extraction of the inoculant (Scheyern, Germany). The natural soil was characterized by an organic C content of 16±0.8 mg g^−1^, N content of 1.76±0.1 mg g^−1^, a C/N-ratio of 9.0±0.4 and a pH value (CaCl_2_) of 6.6±0.1. Prior to spiking, all soils were homogenized and sieved to <2 mm.

The natural soil used for extraction of the inoculant and for the spiking experiment originated from a German research farm (Scheyern; 48°N, 11°E) and no specific permissions were required for collecting. The study did not involve endangered or protected species.

### 2. Spiking experiment

Artificial soils and the natural soil were subjected to the following four spiking treatments: +P, +L, +L+P, unspiked control (Figure 1). Each treatment consisted of four independent replicates. Briefly, phenanthrene spiking was done as follows: a seeding soil (20 mg phenanthrene per gram of soil) was prepared for each artificial soil and the natural soil by adding phenanthrene (≥97%, Merck Schuchardt, Hohenbrunn, Germany) dissolved in acetone (100 mg ml^−1^) to 40 g of soil. Acetone was allowed to evaporate over night to avoid severe changes of the total microbial community. Then, the homogenized seeding soil was mixed to each soil replicate to reach a final phenanthrene concentration of 2 mg g^−1^ of soil [26]. Phenanthrene-unspiked soils received a seeding soil spiked with pure acetone solution. Dried plant litter consisting of maize and potato leaves (1∶1) grown under controlled conditions in the greenhouse was used to conduct the litter amendments on soils (1 wt%). After spiking, the water content was adjusted to a water holding capacity of 60% for each soil composition and treatment. Soils were carefully homogenized in order to save the structure and formed interfaces of matured artificial soils. Incubation was carried out at 14°C, in the dark and at constant water content. Samples were taken on the day of spiking, 7 DAS, 21 DAS, and 63 DAS and subsequently stored at −20°C.

### 3. Extraction and purification of TC-DNA

Extraction of TC-DNA from 0.5 g soil (wet weight) was carried out according to the manufacturer's protocol (FastDNA spin Kit for soil) using FastPrep FP24 bead-beating system (MP Biomedicals, Santa Ana, California). DNA solutions were purified by GeneClean Spin Kit (Qbiogene, Inc., Carlsbad, California). Both steps were followed by controlling the DNA yield on an agarose gel. TC-DNA was kept at −20°C.

### 4. Amplification of bacterial 16S rRNA gene and ITS fragments

All PCRs were performed in a 25 µl reaction with purified and 1∶10 diluted TC-DNA. Total bacterial community was assessed by primers F984GC/R1378 according to Gomes et al. [27]. On samples taken 21 DAS and 63 DAS, nested PCR approaches were carried out for important soil bacterial groups: *Alphaproteobacteria*, *Betaproteobacteria* as described by Gomes et al. [27], *Actinobacteria* [28], and *Pseudomonas* [29]. Diluted amplicons of group-specific PCRs were used as target in PCR with primers F984GC/R1378 to generate the GC-clamp for subsequent DGGE analysis. Fungal communities in artificial soils were studied based on ITS fragments amplified in a nested PCR with primers ITS1F/ITS4 and ITS1F-GC/ITS2 as described by Weinert et al. [30]. Modifications of the conditions of the above PCRs can be found in Babin et al. [24]. Primers with respective references are listed in Table S1.

### 5. DGGE analysis

The microbial community structure was studied by DGGE of amplified 16S rRNA gene or ITS fragments performed in an Ingeny PhorU system (Ingeny, Goes, The Netherlands). Gradient concentrations and DGGE conditions were specified earlier by Weinert et al. [30]. Gels were silver-stained according to Heuer et al. [31].

Extraction of treatment-specific DGGE bands and cloning were carried out as described by Babin et al. [24] and Smalla et al. [32]. Several clones with identical electrophoretic mobility for each band were subjected to sequencing using standard primer SP6 (Macrogen, Amsterdam, The Netherlands). Sequences were cleaned, compared and checked for similarity hits with 16S rRNA gene database entries using Nucleotide BLAST (http://blast.ncbi.nlm.nih.gov/Blast.cgi). Sequences can be retrieved in GenBank under accession numbers KJ145751-KJ145753.

### 6. Quantification of 16S rRNA gene and ITS fragments by qPCR

16S rRNA gene copy numbers were determined by qPCR 5′-nuclease assay with primer and TaqMan probe previously described by Suzuki et al. [33] in a 50 µl reaction volume. Concentrations of reagents and the thermal program can be found in Vogel et al. [25].

Quantification of the fungal ITS fragment was carried out according to the protocol established by Gschwendtner et al. [34] with modifications listed in Vogel et al. [25]. Both quantifications for 16S rRNA gene and ITS fragments were performed in a CFX96 Real-Time System (Biorad, München, Germany). Information on primers and TaqMan probe can be found in Table S1.

### 7. Phenanthrene analysis

The phenanthrene concentration was measured in three replicates per soil and treatment of samples taken on the day of spiking and 21 DAS. Quantification was done according to existing methods [26], [35] with slight modifications as follows: One gram soil was extracted using 5 ml acetone and 5 ml cyclohexane in an ultrasonic bath and an overhead shaker. The final extract was separated with an acetonitrile-water gradient on a C18 column (Luna C18, 100 A, 150×2.0 mm, 3 µm; Phenomenex, Aschaffenburg, Germany). Phenanthrene was detected at 254 nm (UVD 340 S UV detector; Dionex, Sunnyvale, California). External quantification was done with multicomponent standard solution SRM 1647D (National Institute of Standards and Technology, Promochem, Wesel, Germany) at concentrations ranging from 1 to 10 µg ml^−1^. Linearity was excellent (R^2^ = 0.996).

### 8. Statistical analysis

DGGE profiles were compared pairwise using software GelCompar II 6.5 (Applied Maths, Sint-Martens-Latem, Belgium). Pairwise Pearson similarity coefficients were used for the construction of dendrograms based on the unweighted pair group method with arithmetic mean (UPGMA) cluster algorithm and to perform permutation tests for significant differences (d-values) between soil compositions and treatments according to Kropf et al. [36]. The qPCR data and phenanthrene concentrations were subjected to analysis of variance in conjunction with Tukey's HSD test with software R 3.0.2 using package *agricolae*. Multiple factor ANOVA was used to test for significant effects of soil, phenanthrene, and litter. Bacterial 16S rRNA gene and fungal ITS fragment copy numbers were log transformed prior to statistical analysis.

## Results

### 1. The bacterial community structure in long-term matured artificial soils

**Figure 2:**
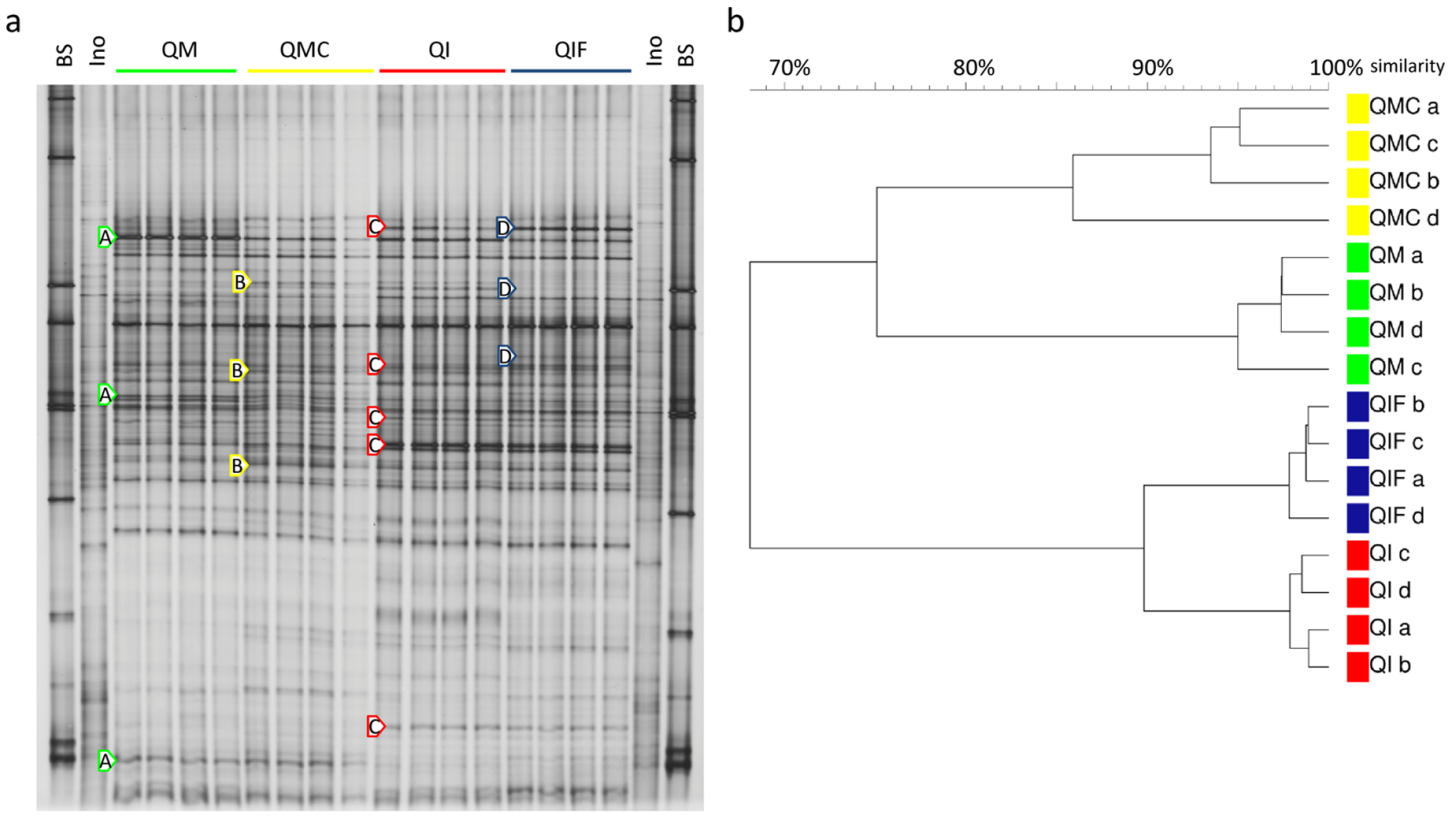
Bacterial communities of long-term matured artificial soils. a) Total bacterial DGGE fingerprints of unspiked control artificial soils (four replicates) sampled on the day of spiking. Arrows mark soil composition-specific populations in QM (bands A), QMC (B), QI (C), and QIF (D). BS-bacterial DGGE standard. Ino-Luvisol inoculant added to soil mixtures at the beginning of incubation. Q-quartz, M-montmorillonite, C-charcoal, I-illite, F-ferrihydrite. b) Corresponding UPGMA cluster analysis.

By DGGE fingerprints, a high similarity of bacterial communities between the four independent replicates of each artificial soil was observed in samples taken on the day of spiking from the control treatment (Figure 2a). Several soil composition-dependent populations were observed which were marked in Figure 2a by arrows. A strong influence of the type of clay mineral, montmorillonite (bands A) and illite (bands C), on the bacterial community was visible as well as several populations with increased or decreased relative abundance due to the presence of charcoal (bands B) or ferrihydrite (bands D), respectively. UPGMA cluster analysis of DGGE fingerprints revealed a clustering according to the type of clay minerals and distinct sub-clusters for artificial soils containing charcoal (QMC) and ferrihydrite (QIF) (Figure 2b). Permutation tests based on Pearson similarity coefficients showed the highest difference between bacterial communities from artificial soils with different clay minerals present (QM vs. QI 30%). Smaller but also significant values were found for the effect of charcoal (QM vs. QMC 18%) and ferrihydrite (QI vs. QIF 8%).

### 2. Response of total bacteria to spiking in long-term matured artificial soils

The effects of +P, +L, +L+P treatments on the total bacterial community structure of the artificial soils were assessed over time. The differences (d-values) between communities in the treatment of interest vs. the control, i.e. without the respective spike, are reported in Table 1.

**Table 1:** Percent difference (d-values) between microbial communities of different spiking treatments for different taxa and different sampling times (21 and 63 days after spiking [DAS]) per soil.

% difference	*Bacteria*			*Betaproteobacteria*		*Actinobacteria*		*Fungi*	
	7 DAS	21 DAS	63 DAS	21 DAS	63 DAS	21 DAS	63 DAS	21 DAS	63 DAS
**QM vs. QM+P**	**9**	**45**	**10**	**1**	**14**	24	**14**	1	2
**QM+L vs. QM+L+P**	**2**	**16**	**10**	**6**	**8**	**13**	**12**	**3**	**7**
**QM vs. QM+L**	**27**	**54**	**18**	**21**	**15**	**43**	**37**	**14**	**17**
**QM+P vs. QM+L+P**	**14**	**33**	**19**	**29**	**15**	**19**	**32**	**19**	**12**
**QMC vs. QMC+P**	**13**	**17**	**23**	**2**	**19**	**21**	**23**	**2**	**6**
**QMC+L vs. QMC+L+P**	1	**14**	**7**	**20**	**26**	**0**	**14**	**5**	**16**
**QMC vs. QMC+L**	**31**	**37**	**28**	**36**	**12**	**28**	**35**	**18**	**21**
**QMC+P vs. QMC+L+P**	**16**	**38**	**16**	**20**	**22**	**9**	**40**	**10**	**14**
**QI vs. QI+P**	**14**	**51**	**27**	3	**27**	**55**	**24**	1	**4**
**QI+L vs. QI+L+P**	**2**	**19**	**21**	**17**	**17**	**47**	**18**	**13**	**26**
**QI vs. QI+L**	**53**	**54**	**30**	**34**	**52**	**30**	**29**	**19**	**13**
**QI+P vs. QI+L+P**	**34**	**33**	**29**	**45**	**36**	**32**	**24**	**12**	5
**QIF vs. QIF+P**	**22**	**42**	**29**	**12**	**36**	**33**	**12**	1	**3**
**QIF+L vs. QIF+L+P**	**3**	17	**13**	4	**7**	**20**	**18**	**8**	**20**
**QIF vs. QIF+L**	**47**	**50**	**37**	**33**	**34**	**38**	**42**	**16**	**21**
**QIF+P vs. QIF+L+P**	**26**	**35**	**33**	**22**	**21**	**24**	**45**	**22**	**40**
**Luv vs. Luv+P**	**8**	**1**	**27**	**18**	**19**	**7**	**5**	**23**	1
**Luv+L vs. Luv+L+P**	**8**	**11**	**20**	**8**	**24**	**10**	**8**	**5**	**12**
**Luv vs. Luv+L**	**24**	**10**	**23**	**9**	**7**	**32**	**15**	**70**	**28**
**Luv+P vs. Luv+L+P**	**29**	**19**	**18**	**19**	7	**20**	**16**	**40**	**24**

D-values were calculated based on pairwise Pearson similarity coefficients for the effect of phenanthrene (+P) in non-litter or litter-amended soils and the effect of litter (+L) in the absence or presence of phenanthrene. Bold numbers indicate significant differences (p<0.05). Q-quartz, M-montmorillonite, C-charcoal, I-illite, F-ferrihydrite, Luv-natural soil (Luvisol).

In samples taken 7 DAS, populations with increased relative abundance as inferred from band intensity due to the litter addition were observed. Some of these litter responders were found in all artificial soils, but others depended on the soil composition (Figure S1, Figure S2). Bacteria in litter-amended artificial soils differed significantly from the controls. Smaller d-values for the litter effect were found in presence of phenanthrene (+L+P vs. +P) compared to the litter effect in phenanthrene-unspiked soils (+L vs. control, Table 1). Overall, lowest d-values for the litter effect were found in soils containing montmorillonite (QM, QMC). Compared to the litter effect, the response of bacteria to phenanthrene was low 7 DAS (Table 1).

**Figure 3:**
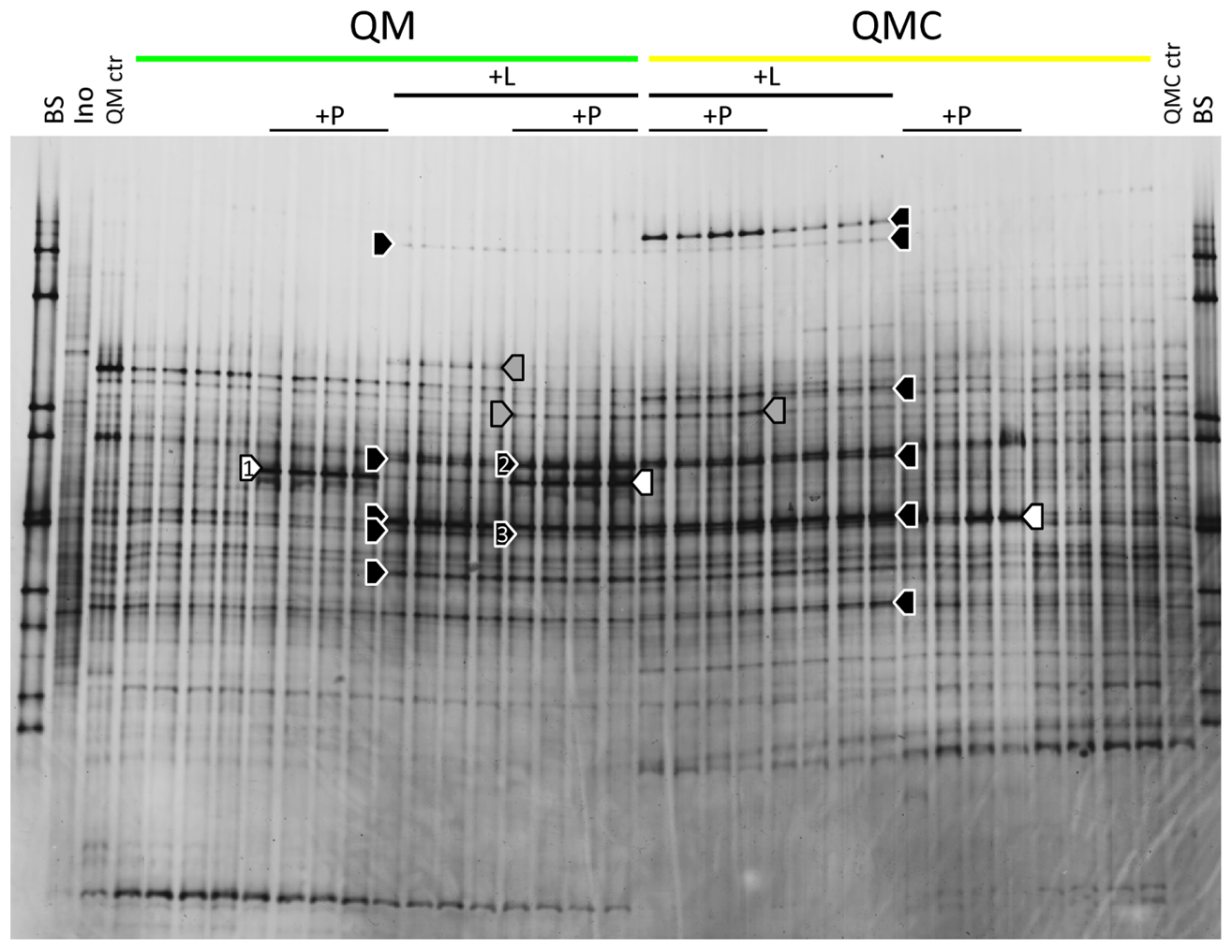
Response of bacterial communities to spiking in QM and QMC soils. DGGE fingerprints of bacterial communities in spiked QM and QMC soils sampled 21 days after spiking (control, phenanthrene (+P), litter (+L), litter and phenanthrene [+L+P]). Arrows mark populations responding to litter (black), phenanthrene (white), litter and phenanthrene (grey). Numbers indicate excised and cloned bands (Table 2). BS-bacterial DGGE standard. Ino-Luvisol inoculant added to soil mixtures at the beginning of incubation. QM/QMC ctr-bacterial community in QM or QMC, respectively, before spiking. Q-quartz, M-montmorillonite, C-charcoal.

Twenty-one DAS, remarkable shifts due to phenanthrene spiking were observed in bacterial communities of all artificial soils (white arrows, Figure 3, Figure S3). In QM+P soils, only one strong responder to phenanthrene was found, which showed 99% similarity to *Arthrobacter crystallopoietes* (band 1 marked by arrow, Figure 3; Table 2). Corresponding bands with similar electrophoretic mobility were detected in QI+P and QIF+P but not in QMC+P soils (DGGE not shown). In QMC+P, another phenanthrene-enhanced population was observed (Figure 3). More shifts in relative abundance in the bacterial community structure due to spiked phenanthrene were found in the QI+P and QIF+P fingerprints (Figure S3). Only a few ferrihydrite-specific responses to phenanthrene were observed (Figure S3). Permutation tests revealed that the response to phenanthrene was as strong as the effect of the litter amendment 21 DAS (d-values ca. 50%) except for QMC soil, in which the difference between the unspiked and phenanthrene-spiked treatment were only 17% (Table 1).

**Table 2:** Tentative phylogenetic affiliation of clones derived from bands excised from bacterial DGGE fingerprints in Figure 3.

Band	Clone (GenBank deposit)	Closest BLAST hits	% Identity	Accession number
1	QMP_1 (KJ145751)	*Arthrobacter crystallopoietes DSM 20117*	99	NR_026189
		*Arthrobacter ramosus DSM 20546*	98	NR_026193
		*Arthrobacter pascens DSM 20545*	98	NR_026191
2	QMPL_2 (KJ145752)	*Stenotrophomonas maltophilia R551-3*	99	NR_074875
		*Stenotrophomonas humi R-32729*	99	NR_042568
		*Stenotrophomosa maltophilia IAM 12423*	99	NR_041577
3	QMPL_3 (KJ145753)	*Arthrobacter sp. FB24*	99	NR_074590.1
		*Arthrobacter humicola KV-653*	99	NR_041546.1
		*Arthrobacter oryzae KV-651*	99	NR_041545.1

GenBank 14-March-2014.

A band with similar electrophoretic mobility as the identified phenanthrene-responding population *Arthrobacter crystallopoietes* (band 1, Figure 3) was also detected in the presence of litter in QM+P+L, QI+P+L, and QIF+P+L soils. Several other populations were found which either increased or decreased in relative abundance due to phenanthrene only in the presence of litter (grey bands, Figure 3, Figure S3). In the presence of litter, the bacterial community shifts in response to phenanthrene were less pronounced compared to the soils without litter amendment (Table 1).

At the sampling time 21 DAS, a remarkable effect of added litter was still observed in all soils in presence and absence of phenanthrene (Table 1). Two populations, which were enhanced in relative abundance in the presence of litter, were excised and cloned from QM+P+L fingerprints (Figure 3). Sequences were affiliated to *Stenotrophomonas maltophilia* (band 2) and *Arthrobacter* sp. (band 3; Table 2). By comparison of bacterial fingerprints of different soils, these two species were identified as responders in all artificial soils to the +L and +L+P treatment except for band 3 in QI+L+P and QIF+L+P soils. Populations with similar electrophoretic mobility compared to band 3 decreased in relative abundance in QI+L+P and QIF+L+P soils compared to QI+L and QIF+L (DGGE not shown).

In order to compare bacterial communities between artificial soils, 16S rRNA gene amplicons of all artificial soils from the same treatment were loaded on one DGGE gel. In samples taken 21 DAS, soil composition-dependent differences between bacteria among soils of the control treatment were observed (Table S2). These differences between artificial soils increased by the +P spiking compared to the control treatment (Table S2). High d-values were found for comparisons between bacteria in QM+P and other artificial soil compositions. The effect of ferrihydrite in litter-amended soils (QI+L vs. QIF+L) was comparable to the control treatment. Spiking of +P, +L, +L+P resulted in higher dissimilarities between bacteria in QM vs. QMC soils than in the control treatment.

In QI, QIF, and QM soils sampled 63 DAS, shifts in bacterial communities caused by spiking were similar to the shifts in samples of the respective artificial soil taken 21 DAS. In contrast, bacteria in QMC soils showed 63 DAS a stronger response to phenanthrene by pronounced shifts which were similar in their electrophoretic mobility to phenanthrene-responding populations in QM soils (Figure S4, Table 1).

### 3. Response of bacterial groups to spiking in long-term matured artificial soils

Cluster analysis of *Alphaproteobacteria* and *Pseudomonas* communities in samples taken 21 DAS showed effects of the soil composition and the litter addition, while the phenanthrene influence on these taxa was small (Figure S5). *Betaproteobacteria* in soils sampled 21 DAS were affected by the soil composition and litter amendment. A response of *Betaproteobacteria* to phenanthrene was only seen in the +L+P treatment (except for QIF; Table 1). Sixty-three DAS, more changes in betaproteobacterial fingerprints were observed due to phenanthrene compared to 21 DAS especially in QMC+P, QI+P, and QIF+P soils (Table 1). In QMC soils sampled 21 DAS, different shifts due to spiked phenanthrene were observed in actinobacterial fingerprints compared to QM soils (white arrows, Figure S6). *Actinobacteria* in QI and QIF soils responded in a similar way to phenanthrene, but shifts were more pronounced in QI soils (Table 1). A few soil composition and litter responders were found among the *Actinobacteria* (Figure S6, Table 1). The QMC soil exhibited clearly more treatment-responding actinobacterial populations than the QM soils at sampling time 63 DAS compared to 21 DAS (Table 1).

### 4. Response of fungal communities to spiking in long-term matured artificial soils

**Figure 4:**
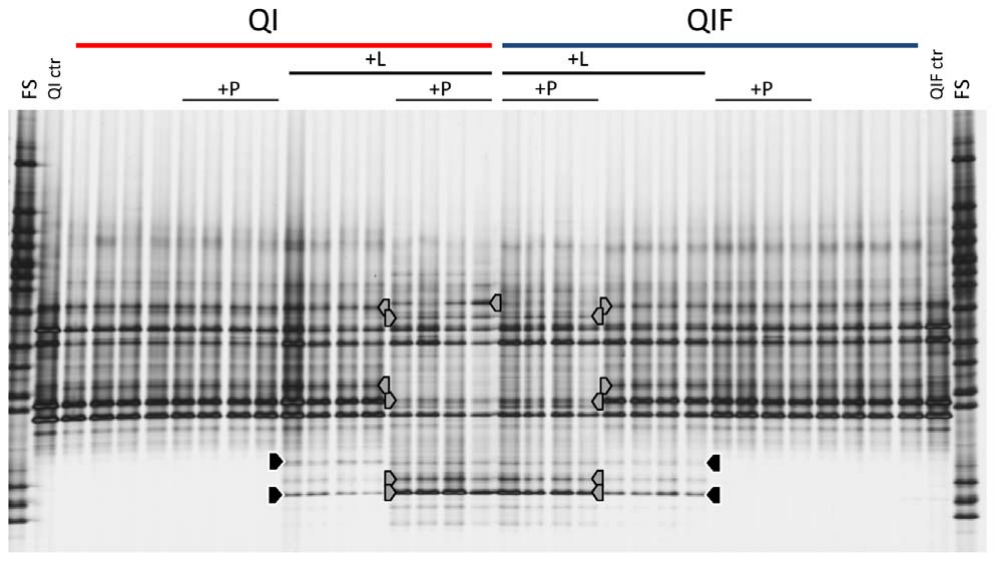
Response of fungal communities to spiking in QI and QIF soils. DGGE fingerprints of fungal communities in spiked QI and QIF soils sampled 63 days after spiking (control, phenanthrene (+P), litter (+L), litter and phenanthrene [+L+P]). Arrows mark populations responding to litter (black), litter and phenanthrene (grey). FS-fungal DGGE standard. QI/QIF ctr-fungal community in QI or QIF, respectively, before spiking. Q-quartz, I-illite, F-ferrihydrite.

At sampling time 21 DAS, the difference of fungal DGGE fingerprints between QI and QIF control soils and between QM and QMC control soils accounted for 9% and 20%, respectively. In all artificial soil samples taken 21 DAS and 63 DAS, weak fungal responses to +L addition were observed (black arrows, Figure 4, Figure S7), whereas no effects of +P were detected (Table 1). A profound effect on fungi was observed in all artificial soils for the +L+P spiking in samples taken 21 DAS, which even increased up to sampling time 63 DAS (Table 1). The +L+P treatment caused the detection of a few additional bands but also the disappearance of bands that were strongly abundant in the corresponding control. The shifts in fungal communities due to +L+P were similar in QI+L+P and QIF+L+P soils (grey bands, Figure 4). As for bacteria, the presence of charcoal also influenced the fungal response to +L+P treatment in QMC soils compared to QM by the appearance of additional responder bands (Figure S7).

### 5. Response of microbial communities to spiking in the natural soil

The bacterial community of the natural soil (Luv) revealed a different and more complex pattern in DGGE analysis than the artificial soils (Figure S8). Bacterial communities in the natural soil responded also to the different treatments, but the shifts were less pronounced, as indicated by lower d-values for the effect of spiking compared to the treatment effects in artificial soils (Figure S8, Table 1). The common responders of artificial soils (bands 1–3, Figure 3) were not found in the spiked natural soil. Twenty-one DAS, there was mainly a response of bacteria to the litter addition detectable in the natural soil. The effect of phenanthrene was retarded and only clearly visible in samples taken 63 DAS (Figure S8, Table 1). Taxon-specific DGGE analysis revealed that typical phenanthrene responders in the natural soil belonged to the *Betaproteobacteria* group (Table 1).

Fungal communities in the natural soil were also more complex and more heterogeneous than fungi in artificial soils. A significant response of fungi to +L was observed, especially 21 DAS, whereas shifts due to +L+P were not as pronounced as found in artificial soils (Table 1).

### 6. Quantification of bacterial 16S rRNA gene and fungal ITS fragments

**Figure 5:**
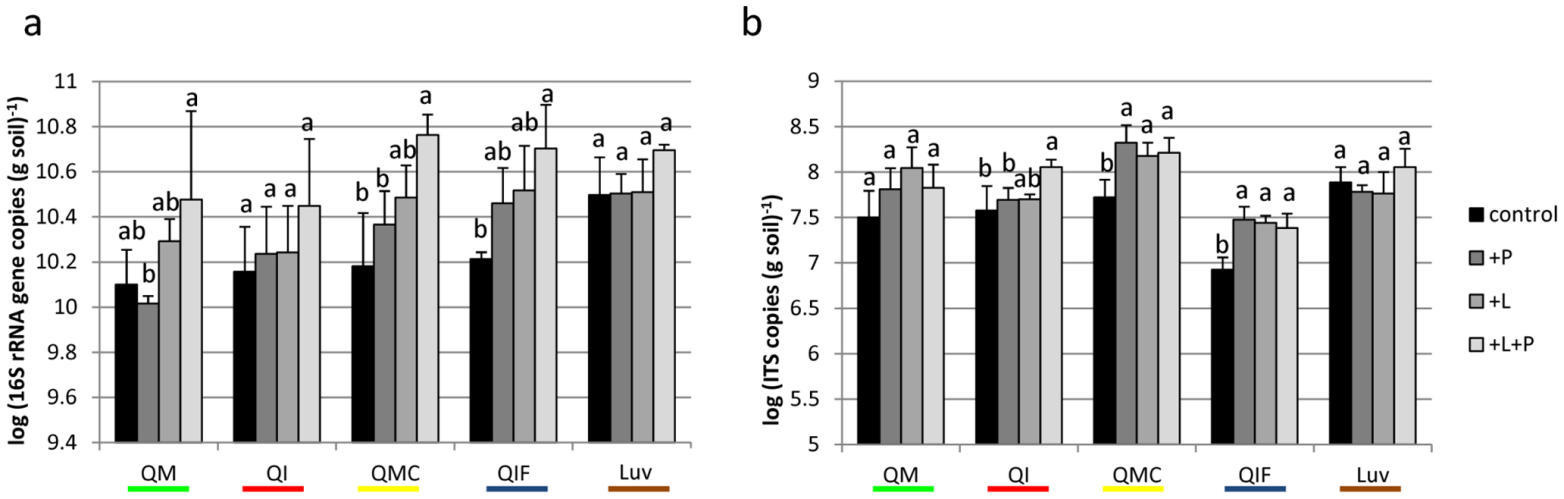
Quantification of 16S rRNA gene and ITS fragments in spiked soils. The qPCR analysis of (a) bacterial 16S rRNA gene copy numbers in samples taken 21 days after spiking (DAS) and (b) of fungal ITS fragment copy numbers in samples taken 63 DAS in the following treatments: unspiked (control), phenanthrene (+P), litter (+L), litter and phenanthrene (+L+P). Different letters within one soil composition mark a significant difference between treatments (Tukey test, p<0.05). Bars indicate standard deviation of four replicates. Q-quartz, M-montmorillonite, C-charcoal, I-illite, F-ferrihydrite, Luv-natural soil (Luvisol).

The amount of 16S rRNA genes was determined in artificial soils and the natural soil sampled 21 DAS (Figure 5a). In the control treatment, gene copy numbers among artificial soils were similar and ranged from 1.3×10^10^ to 1.6×10^10^. A higher abundance was detected in the natural soil (Luv; 3.2×10^10^). Phenanthrene caused a slight increase of 16S rRNA gene copies in QI+P, QMC+P, and QIF+P. Except for QI+L soils, litter addition resulted in clearly enhanced copy numbers. The highest numbers were found for all soils in the +L+P treatment. 16S rRNA gene copy numbers in the natural soil were relatively stable and were only enhanced in +L+P treatment. Analysis of variance revealed highly significant effects for litter (p<0.001), phenanthrene (p<0.001), and soil (p<0.001).

Fungal ITS fragments were quantified in differently treated artificial soils and the natural soil sampled 63 DAS (Figure 5b), since most pronounced shifts in the DGGE fingerprints were observed at this sampling time. Abundance of ITS fragments in artificial soils was in the range of the natural soil (Luv) in all treatments (3.2×10^7^ to 2.1×10^8^), except for soil QIF which showed significantly lower numbers (8.4×10^6^ to 3×10^7^). In all artificial soils, ITS copy numbers increased by spiking treatments compared to the control. The abundance of ITS fragments in the natural soil (Luv) was relatively stable over all treatments. Spiking (p<0.001), soil (p<0.001), and the interaction of both factors (p = 0.01) did significantly influence the ITS copy numbers.

### 7. Phenanthrene concentrations in spiked soils

Phenanthrene concentrations in spiked soils sampled on the day of spiking and 21 DAS are presented in Table 3. In unspiked control treatments, negligible amounts of phenanthrene were detected (<0.01 mg g^−1^ of soil; data not shown). Except for soil QM+P, slightly less phenanthrene than the spiked amount of 2 mg g^−1^ was recovered from all soils without litter amendment sampled on the day of spiking. In two replicates of soil QM+P, instead, a considerably lower concentration of phenanthrene (0.71 and 0.66 mg g^−1^) was detected. Also in all +L+P soils sampled on the day of spiking, significantly lower concentrations were recovered compared to soils without litter (p<0.001). Twenty-one DAS, the phenanthrene concentrations in soils without the litter amendment decreased except for the two replicates of soil QM+P and one replicate of the natural soil (Luv+P) which showed higher amounts compared to the day of spiking. The lowest phenanthrene concentrations 21 DAS were found in QI+P and QIF+P soils. However, no significant differences were found between soils. The amount of phenanthrene in +L+P soils sampled 21 DAS was slightly lower compared to the concentrations measured on the day of spiking in +L+P soils. Soils containing illite had also the lowest amount of phenanthrene in +L+P treatment 21 DAS.

**Table 3:** Phenanthrene concentrations in mg g^−1^ soil determined in three replicates per soil composition in the phenanthrene (+P), litter and phenanthrene (+L+P) treatments on the day of spiking and 21 days after spiking (DAS).

P [mg g^−1^]	+P		+L+P	
	day of spiking	21 DAS	day of spiking	21 DAS
**QM**	0.71	2.40	0.74	0.40
	1.76	1.08	0.49	0.68
	0.66	1.15	0.88	0.64
**QMC**	1.71	1.41	1.02	0.56
	1.63	1.53	0.75	0.54
	1.74	1.51	0.87	0.66
**QI**	1.36	0.89	0.92	0.26
	1.86	0.46	0.76	0.22
	1.78	0.81	0.66	0.30
**QIF**	1.14	0.33	0.85	0.27
	1.80	0.87	0.78	0.51
	1.74	1.33	0.60	0.45
**Luv**	1.45	0.77	0.56	0.73
	1.69	1.22	0.78	0.68
	1.68	1.72	0.90	0.77

Q-quartz, M-montmorillonite, C-charcoal, I-Illite, F-ferrihydrite, Luv-natural soil (Luvisol).

## Discussion

### 1. Artificial soils exhibited soil composition-driven microbial communities

Amplicon-based fingerprinting analysis of bacteria and fungi in unspiked control artificial soils confirmed that the structure of the microbial communities was driven by soil composition. In agreement with Vogel et al. [25], who described the microbiota establishment in artificial soils incubated up to 842 days, we observed that clay minerals strongly influenced bacterial communities. Charcoal and ferrihydrite shaped bacterial communities to a lesser extent. However, Vogel et al. [25] did not find an effect of soil components on fungi in long-term incubated artificial soils as observed in the present study. It is likely that the preparation of soils for the spiking treatments destroyed existing hyphae and aggregates so that soil components could re-induce their effects on fungi. In conclusion, microbial communities differed significantly among soil compositions incubated for 842 days.

### 2. Spiking changed bacterial communities in artificial soils

The litter addition resulted in rapid changes in the bacterial community composition in artificial soils as indicated by DGGE. The control fingerprint of 16S rRNA genes amplified from litter TC-DNA showed no similarities with bacteria in artificial soils (Figures S2–S4). We therefore suggest that observed responders were not introduced by the litter amendment, but were due to a proliferation of artificial soil bacteria in response to the litter addition. *Stenotrophomonas maltophilia* and *Arthrobacter* were identified as potential litter responders that thrived equally well in all litter-amended artificial soils. Besides being a typical soil bacterium, *Arthrobacter* species are known for their tolerance to extreme conditions and their broad nutrient spectrum [37]. The trophic versatility of the *Stenotrophomonas* genus ranges from pathogenicity to humans, plant growth promoting and biocontrol properties, and the production of bioactive substances, to the ability to degrade a wide range of organic compounds, including PAHs [38]. The latter fact might also have favored their establishment in +L+P treated soils. Furthermore, the litter addition caused a strong increase of bacterial 16S rRNA gene copy numbers in all soils (except QI+L). It is likely that only recalcitrant, polymerized organics remained of the two sterile manure inputs that artificial soils received during more than two years of maturation and that phenanthrene and litter thus represented easily available nutrient sources for the microbiota. The degradation of phenanthrene is supported by the lower phenanthrene concentrations measured, and the increase in 16S rRNA gene copy numbers found in all +P soils except QM+P (see section 4 for further discussion). Furthermore, phenanthrene resulted in several shifts in the bacterial community composition of samples taken 21 DAS and 63 DAS compared to the day of spiking. The high similarity of the sequenced DGGE band to *Arthrobacter crystallopoietes* provided further evidence for the proliferation of species potentially capable of degrading aromatic substances. *Arthrobacter crystallopoietes* has been reported to degrade aromatic compounds in soil [39], [40] and to survive under long-term starvation conditions [41], which is consistent with our assumption of nutrient-limiting conditions in artificial soils [25]. Group-specific DGGE analyses suggested that *Actinobacteria* responded most to phenanthrene in all artificial soils which is in accordance with the phenanthrene response in one-year matured artificial soils [24]. However, in contrast to Babin et al. [24], we could not detect an increase in the dioxygenase gene abundance in +P and +L+P treated long-term matured artificial soils (data not shown). To conclude, spiking of long-term matured artificial soils with phenanthrene and litter resulted in significant shifts in the bacterial community structure.

### 3. Litter addition did not stimulate the response of bacterial communities to phenanthrene in artificial soils

We observed that the +L+P spiking to artificial soils resulted in clearly increased 16S rRNA gene copy numbers indicating that bacterial activity was triggered by this treatment. Several studies have demonstrated positive effects of compost, fertilizer, manure, and poultry litter addition on the degradation of contaminants in polluted soils [42], [43], [44], [45]. We observed that bacterial community composition of artificial soils was significantly influenced by the +L+P spiking but to a lesser extent than by the +P spiking. Some litter-induced phenanthrene responders were observed, e.g. *Betaproteobacteria*, but, unfortunately, firm conclusions on the stimulatory effect of litter on degradation of phenanthrene cannot be drawn due to the low recovery of phenanthrene in presence of litter. In contrast to other studies, we added phenanthrene and litter simultaneously and the ground litter likely offered a high sorption surface. PAHs were previously shown to have a high affinity to soil OM [11]. We assume that phenanthrene was likely less accessible to bacteria in the +L+P than in the +P treatment. This assumption is supported by the lack of enhanced abundance of IncP-9 plasmids in +L+P spiked artificial soils (data not shown), even though nutrient sources in soils are generally considered to be microbial hotspots conducive to horizontal gene transfer events. Furthermore, we observed major shifts in *Pseudomonas* and *Alphaproteobacteria* fingerprints due to litter. It is possible that copiotrophic bacteria in artificial soils outcompeted phenanthrene degraders in +L+P treatment [46]. Hence, to get more insights into the influence of the +L+P spiking, further analyses of the microbial community changes (e.g. by pyrosequencing) and the metabolic activity are needed.

### 4. Soil composition controlled bacterial response to spiking

The spiking of +P, +L, +L+P to long-term matured artificial soils enabled us to study the response of the microbiota in a soil system established as a function of the soil composition. We observed similar and completely soil composition-specific responses to phenanthrene spiking confirming the results of our one year artificial soil study [24]. Similar responses among different bacterial communities indicate that comparable conditions in different artificial soils allowed the establishment of common taxa. However, it was more striking that phenanthrene even strengthened the difference between the microbiota of artificial soils, especially for soils containing different clay minerals. Correspondingly, other authors [12], [13], [26] have reported that contamination did not result in converging of microbial communities in different soil types. The different responses observed in the present study can be caused by the soil-specific initial microbial communities present in artificial soils before spiking treatments allowing or preventing the establishment of certain populations. These different initial microbial communities are caused by an establishment in an environment solely driven by the soil composition. That is why we conclude that the soil composition controlled the bacterial response to spiking. However, further analyses are needed to determine whether and to what extent indirect effects, i.e. the different interfaces established during incubation of artificial soils or a complex interaction between all soil components might have contributed to the observed differences.

Our results consistently showed that bacteria in QM soils responded less to phenanthrene compared to soils containing illite (QI, QIF). In a previous study of these artificial soils it was shown that the organic carbon concentration in QM soils was slightly higher compared to other soils [25] which might have resulted in sequestration of phenanthrene to the soil OM and its reduced bioavailability [5], [9]. Furthermore, Vogel et al. [25] reported previously that QM and QMC soils incubated over a long-term period contained more aggregates than soils with illite. It is known that the biodegradation of PAHs is restricted in aggregates [2], [47], [48], [49]. Montmorillonite is an expandable clay mineral with a high specific surface area which makes possible that phenanthrene was separated from bacterial cells by surface sorption or entrapment in small pores [10], [50]. A lower degradation rate of phenanthrene in the presence of expandable clays has been shown in previous studies [51], [52]. However, clay minerals also have previously been reported to have a positive influence on the biodegradation efficiency, e.g. by enabling the degradation of the bound pollutant by adhesion of microbial cells [51], [53], facilitating transformation of chemical compounds [54], and stimulating microbial growth [55]. Our data suggest that soils containing illite might favor microbial degradation in this respect compared to montmorillonite. Unfortunately, this assumption cannot be proven, as the phenanthrene concentrations differed strongly among QM replicates. It is possible that mixing was not sufficient to prevent the formation of phenanthrene hotspots.

Besides clay minerals, we observed an effect of the charcoal component on the microbial response to spiking. Bacterial communities in QMC soils showed a delayed response to phenanthrene. Charcoal and soil OM in general are known to be the principal sorption sites for PAHs [11]. Along with the slightly higher phenanthrene concentrations in QMC compared to QI and QIF soils sampled 21 DAS, it can be assumed that only a slow desorption of phenanthrene from charcoal took place, which would be only degraded over a longer term than the incubation time with spikes used in this study [10].

### 5. Combined spiking of phenanthrene and litter increased effects on fungal communities

Fungal communities were almost insusceptible to the +P or +L spiking, whereas the combined +L+P spiking strongly changed the fungal community composition. Ligninolytic fungi are known to express enzymes with low substrate specificity that also makes them capable of degrading PAHs [7], [56]. We suggest that some of the responsive fungi observed in artificial soils were favored by co-metabolism of phenanthrene, after enzyme expression was induced under nutrient-rich conditions as previously shown for several fungal strains [57], [58].

### 6. Natural soil microbiota less responsive to spiking

Artificial soils incubated for more than two years were previously shown to develop to soil-like systems [25]. In the present study, we subjected these matured artificial soils and a natural soil to similar spiking treatments in order to compare the response of the microbiota. Since we were aware of several missing processes when incubating soils under laboratory conditions (e.g. freezing, thawing, transport, OM input), we were not surprised to observe a number of differences between the microbial responses. For instance, the lower amount of OM in artificial soils compared to the natural soil might have likely been an important factor resulting in higher bioavailability of phenanthrene. Furthermore, we suggest that the less pronounced response of natural soil microbial communities to spiking compared to artificial soils is caused by the higher diversity in the natural soil exhibiting a greater potential to compensate perturbations [59], [60].

## Conclusion

In the present study, we showed that soil composition and presence of charcoal matter by influencing the composition and the response of microbial communities to spiked phenanthrene and litter. We suggest that the soil mineral composition affects the formation of biogeochemical interfaces and of a soil-specific microbiota that control the response to introduced organic substrates. The artificial soil approach may therefore represent a valuable basis for understanding and integrating additional parameters to unravel the complex soil interaction network, and to improve current bioremediation strategies. However, further research is needed to disentangle the single role of the microbiota and the physical soil environment for the fate of pollutants.

## Supporting Information

Figure S1 **Response of bacterial communities to spiking in QM and QMC soils 7 days after spiking.** DGGE fingerprints of bacterial communities in spiked QM and QMC soils sampled 7 days after spiking (control, phenanthrene (+P), litter (+L), litter and phenanthrene [+L+P]). Black arrows mark populations responding to litter. BS-bacterial DGGE standard. Q-quartz, M-montmorillonite, C-charcoal.
(PDF)

Figure S2 **Response of bacterial communities to spiking in QI and QIF soils 7 days after spiking.** DGGE fingerprints of bacterial communities in spiked QI and QIF soils sampled 7 days after spiking (control, phenanthrene (+P), litter (+L), litter and phenanthrene [+L+P]). Black arrows mark populations responding to litter. BS-bacterial DGGE standard. Litter ctr-control fingerprint of litter used for amendments. Q-quartz, I-illite, F-ferrihydrite.
(PDF)

Figure S3 **Response of bacterial communities to spiking in QI and QIF soils 21 days after spiking.** DGGE fingerprints of bacterial communities in spiked QI and QIF soils sampled 21 days after spiking (control, phenanthrene (+P), litter (+L), litter and phenanthrene [+L+P]). Arrows mark populations responding to litter (black), phenanthrene (white), litter and phenanthrene (grey). BS-bacterial DGGE standard. Ino-Luvisol inoculant added to soil mixtures at the beginning of incubation. QI/QIF ctr-bacterial community in QI or QIF, respectively, before spiking. Litter ctr-control fingerprint of litter used for amendments. Q-quartz, I-illite, F-ferrihydrite.
(PDF)

Figure S4 **Response of bacterial communities in QM and QMC soils 63 days after spiking.** DGGE fingerprints of bacterial communities in spiked QM and QMC soils sampled 63 days after spiking (control, phenanthrene (+P), litter (+L), litter and phenanthrene [+L+P]). Arrows mark populations responding to litter (black), phenanthrene (white), litter and phenanthrene (grey). BS-bacterial DGGE standard. QM/QMC ctr-bacterial community in QM or QMC, respectively, before spiking. Litter ctr-control fingerprint of litter used for amendments. Q-quartz, M-montmorillonite, C-charcoal.
(PDF)

Figure S5 **Dendrograms of** ***Alphaproteobacteria*** **and** ***Pseudomonas*** **communities in artificial soils.** UPGMA cluster analysis of DGGE fingerprints of taxon-specific 16S rRNA gene amplicons from artificial soils a) QM and QMC b) QI and QIF sampled 21 days after spiking (control, phenanthrene (+P), litter (+L), litter and phenanthrene [+L+P]). Q-quartz, M-montmorillonite, C-charcoal, I-illite, F-ferrihydrite.
(PDF)

Figure S6 **Response of** ***Actinobacteria*** **in QM and QMC soils to spiking.** DGGE fingerprints of actinobacterial communities in spiked QM and QMC soils sampled 21 days after spiking (control, phenanthrene (+P), litter (+L), litter and phenanthrene [+L+P]). Arrows mark populations responding to litter (black), phenanthrene (white). BS-bacterial DGGE standard. Ino-Luvisol inoculant added to soil mixtures at the beginning of incubation. Q-quartz, M-montmorillonite, C-charcoal.
(PDF)

Figure S7 **Response of fungal communities to spiking in QM and QMC soils.** DGGE fingerprints of fungal communities in spiked QM and QMC soils sampled 63 days after spiking (control, phenanthrene (+P), litter (+L), litter and phenanthrene [+L+P]). Arrows mark populations responding to litter (black), litter and phenanthrene (grey). FS-fungal DGGE standard. QM/QMC ctr-fungal community in QM or QMC, respectively, before spiking. Q-quartz, M-montmorillonite, C-charcoal.
(PDF)

Figure S8 **Response of bacterial communities to spiking in the natural soil.** DGGE fingerprints of bacterial communities in the spiked natural Luvisol soil (Luv) sampled 63 days after spiking (control, phenanthrene (+P), litter (+L), litter and phenanthrene [+L+P]). Arrows mark populations responding to litter (black), phenanthrene (white). BS-bacterial DGGE standard. Luv ctr-bacterial community in the natural soil before spiking.
(PDF)

Table S1 **Primers and probe used in the study.**
(PDF)

Table S2 **Percent difference (d-values) between bacterial communities of different artificial soils per treatment (control, phenanthrene (+P), litter (+L), litter and phenanthrene [+L+P]) 21 days after spiking.** D-values were calculated based on pairwise Pearson similarity coefficients and were significant for all comparisons (p<0.05). Q-quartz, M-montmorillonite, C-charcoal, I-illite, F-ferrihydrite.
(PDF)
