# Supplementary material for: Soil Mineral Composition Matters: Response of Microbial Communities to Phenanthrene and Plant Litter Addition in Long-Term Matured Artificial Soils
Source: PLoS One. 2014 Sep 15;9(9):e106865. doi: 10.1371/journal.pone.0106865 (PMC4164357; doi:10.1371/journal.pone.0106865)
Supplement: Figure S1 — Response of bacterial communities to spiking in QM and QMC soils 7 days after spiking. DGGE fingerprints of bacterial communities in spiked QM and QMC soils sampled 7 days after spiking (control, phenanthrene (+P), litter (+L), litter and phenanthrene [+L+P]). Black arrows mark populations responding to litter. BS-bacterial DGGE standard. Q-quartz, M-montmorillonite, C-charcoal. (PDF) [file pone.0106865.s001.pdf]

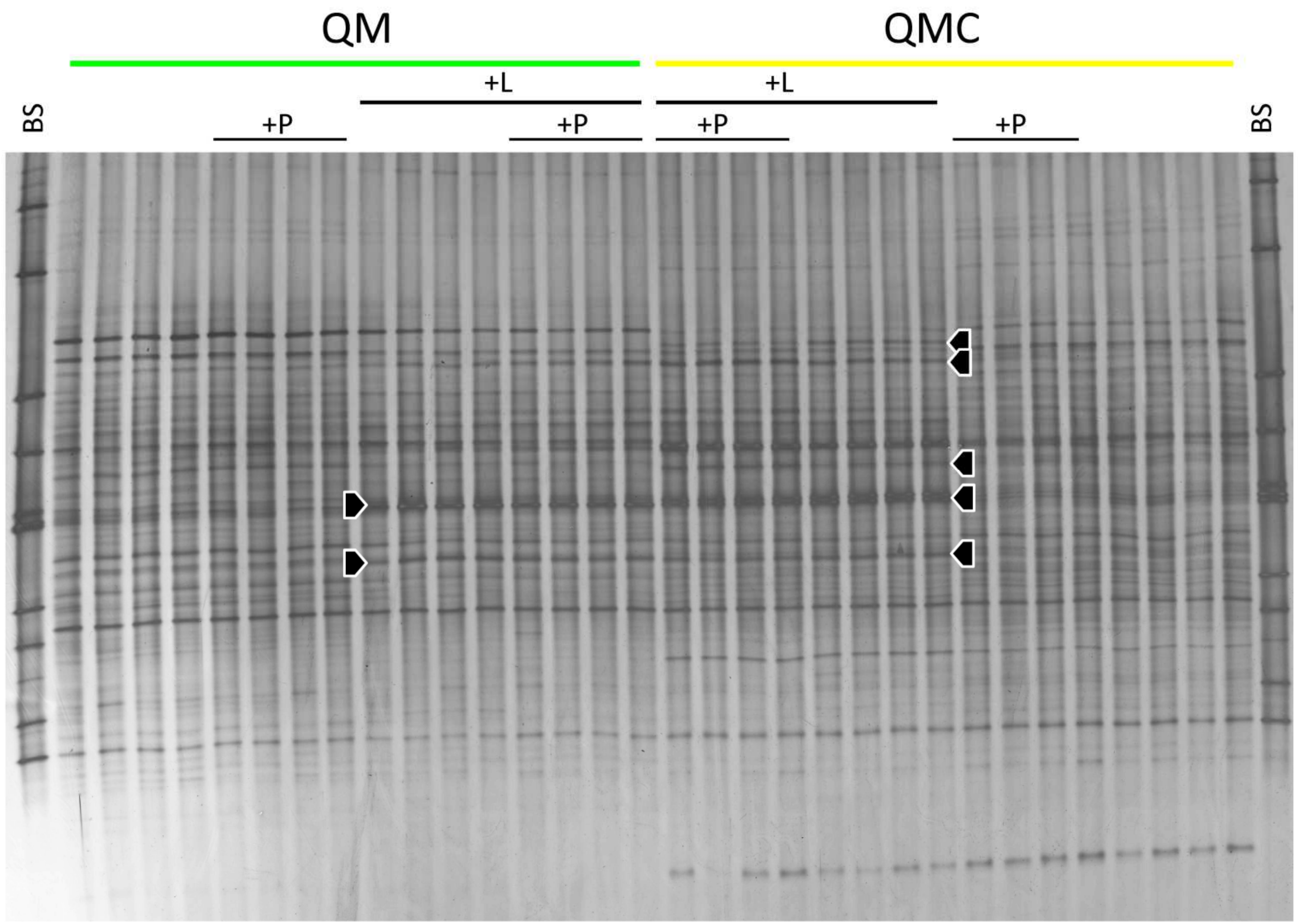

Figure S1. **Response of bacterial communities to spiking in QM and QMC soils 7 days after spiking.** DGGE fingerprints of bacterial communities in spiked QM and QMC soils sampled 7 days after spiking (control, phenanthrene (+P), litter (+L), litter and phenanthrene [+L+P]). Black arrows mark populations responding to litter. BS-bacterial DGGE standard. Q-quartz, M-montmorillonite, C-charcoal.
