# Supplementary material for: Soil Mineral Composition Matters: Response of Microbial Communities to Phenanthrene and Plant Litter Addition in Long-Term Matured Artificial Soils
Source: PLoS One. 2014 Sep 15;9(9):e106865. doi: 10.1371/journal.pone.0106865 (PMC4164357; doi:10.1371/journal.pone.0106865)
Supplement: Figure S2 — Response of bacterial communities to spiking in QI and QIF soils 7 days after spiking. DGGE fingerprints of bacterial communities in spiked QI and QIF soils sampled 7 days after spiking (control, phenanthrene (+P), litter (+L), litter and phenanthrene [+L+P]). Black arrows mark populations responding to litter. BS-bacterial DGGE standard. Litter ctr-control fingerprint of litter used for amendments. Q-quartz, I-illite, F-ferrihydrite. (PDF) [file pone.0106865.s002.pdf]

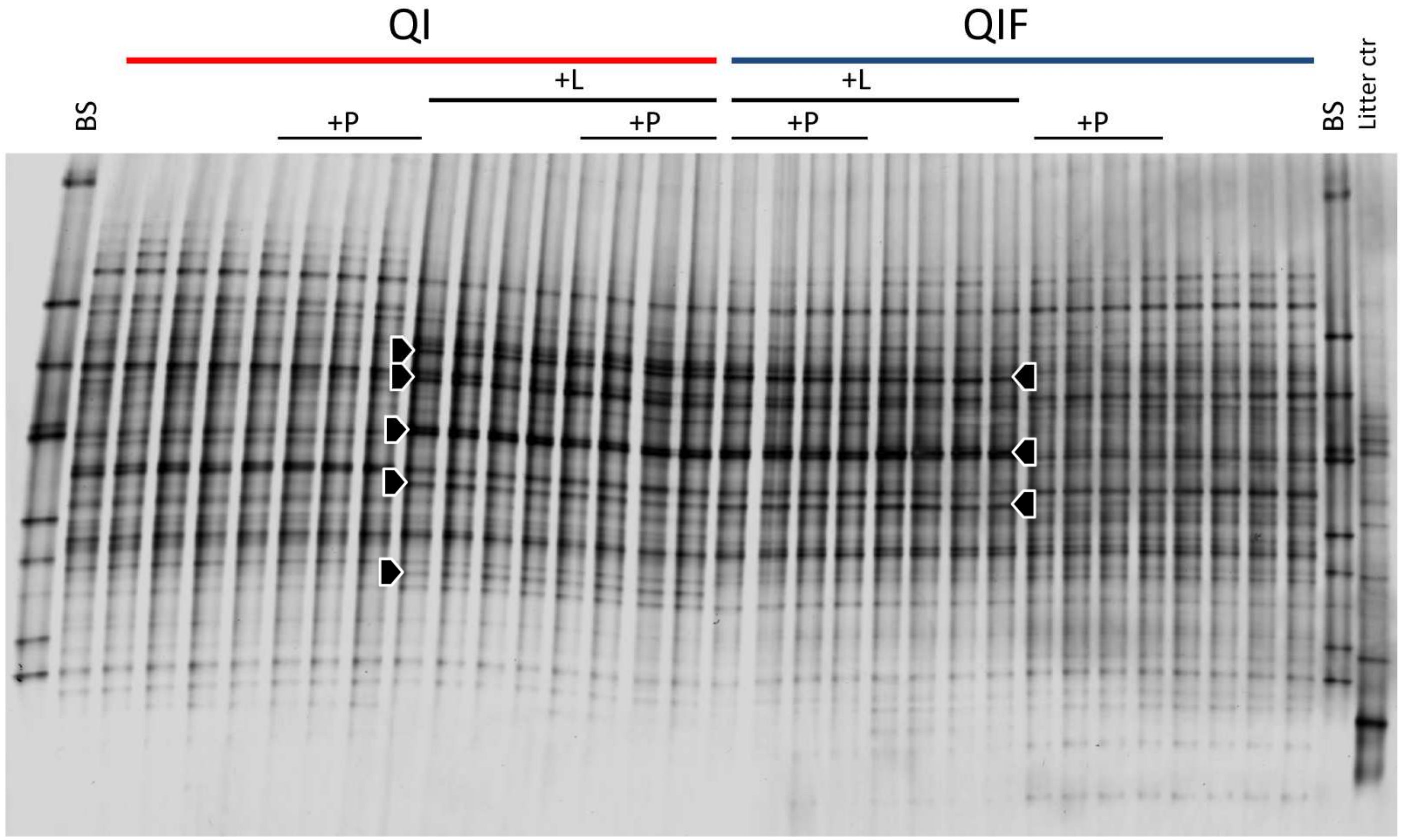

Figure S2. **Response of bacterial communities to spiking in QI and QIF soils 7 days after spiking.** DGGE fingerprints of bacterial communities in spiked QI and QIF soils sampled 7 days after spiking (control, phenanthrene (+P), litter (+L), litter and phenanthrene [+L+P]). Black arrows mark populations responding to litter. BS-bacterial DGGE standard. Litter ctr-control fingerprint of litter used for amendments. Q-quartz, I-illite, F-ferrihydrite.
