# Supplementary material for: Soil Mineral Composition Matters: Response of Microbial Communities to Phenanthrene and Plant Litter Addition in Long-Term Matured Artificial Soils
Source: PLoS One. 2014 Sep 15;9(9):e106865. doi: 10.1371/journal.pone.0106865 (PMC4164357; doi:10.1371/journal.pone.0106865)
Supplement: Figure S3 — Response of bacterial communities to spiking in QI and QIF soils 21 days after spiking. DGGE fingerprints of bacterial communities in spiked QI and QIF soils sampled 21 days after spiking (control, phenanthrene (+P), litter (+L), litter and phenanthrene [+L+P]). Arrows mark populations responding to litter (black), phenanthrene (white), litter and phenanthrene (grey). BS-bacterial DGGE standard. Ino-Luvisol inoculant added to soil mixtures at the beginning of incubation. QI/QIF ctr-bacterial community in QI or QIF, respectively, before spiking. Litter ctr-control fingerprint of litter used for amendments. Q-quartz, I-illite, F-ferrihydrite. (PDF) [file pone.0106865.s003.pdf]

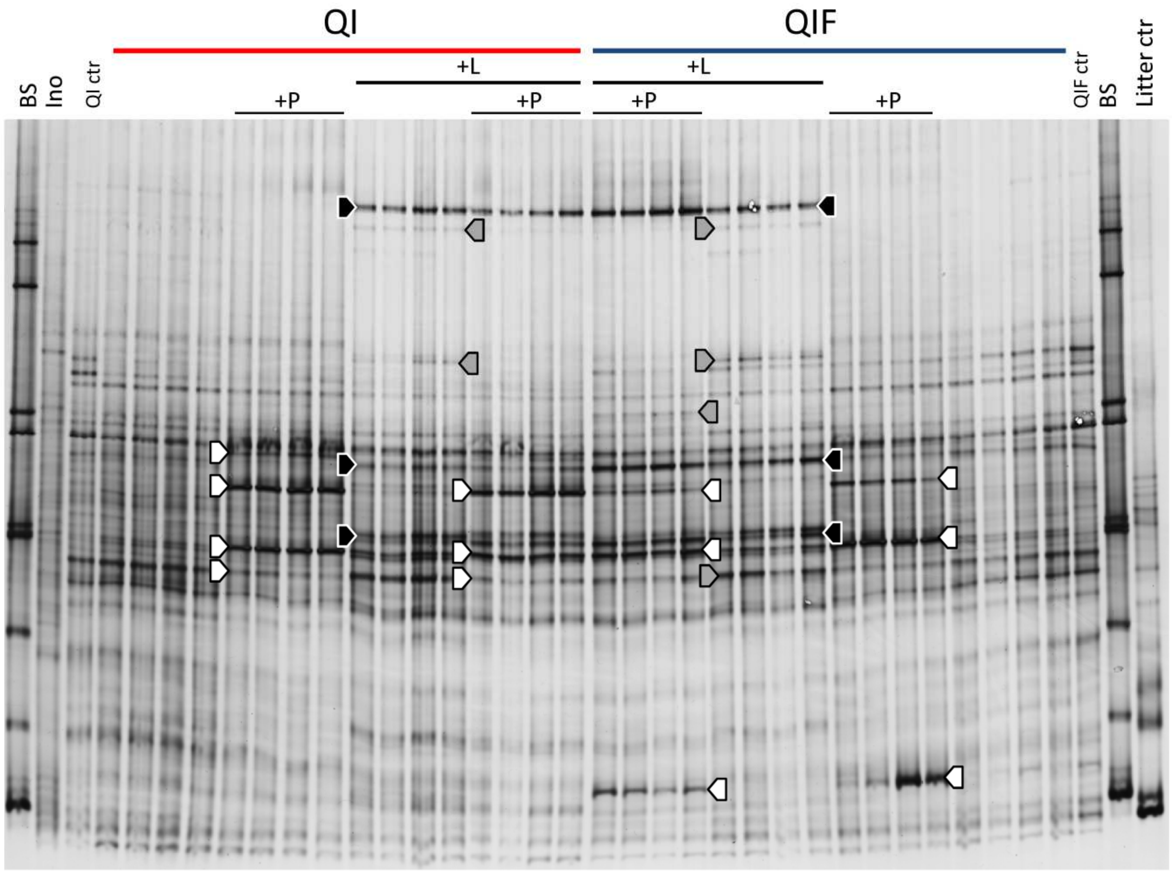

Figure S3. **Response of bacterial communities to spiking in QI and QIF soils 21 days after spiking.** DGGE fingerprints of bacterial communities in spiked QI and QIF soils sampled 21 days after spiking (control, phenanthrene (+P), litter (+L), litter and phenanthrene [+L+P]). Arrows mark populations responding to litter (black), phenanthrene (white), litter and phenanthrene (grey). BS-bacterial DGGE standard. Ino-Luvisol inoculant added to soil mixtures at the beginning of incubation. QI/QIF ctr-bacterial community in QI or QIF, respectively, before spiking. Litter ctr-control fingerprint of litter used for amendments. Q-quartz, I-illite, F-ferrihydrite.
