# Supplementary material for: Soil Mineral Composition Matters: Response of Microbial Communities to Phenanthrene and Plant Litter Addition in Long-Term Matured Artificial Soils
Source: PLoS One. 2014 Sep 15;9(9):e106865. doi: 10.1371/journal.pone.0106865 (PMC4164357; doi:10.1371/journal.pone.0106865)
Supplement: Figure S4 — Response of bacterial communities in QM and QMC soils 63 days after spiking. DGGE fingerprints of bacterial communities in spiked QM and QMC soils sampled 63 days after spiking (control, phenanthrene (+P), litter (+L), litter and phenanthrene [+L+P]). Arrows mark populations responding to litter (black), phenanthrene (white), litter and phenanthrene (grey). BS-bacterial DGGE standard. QM/QMC ctr-bacterial community in QM or QMC, respectively, before spiking. Litter ctr-control fingerprint of litter used for amendments. Q-quartz, M-montmorillonite, C-charcoal. (PDF) [file pone.0106865.s004.pdf]

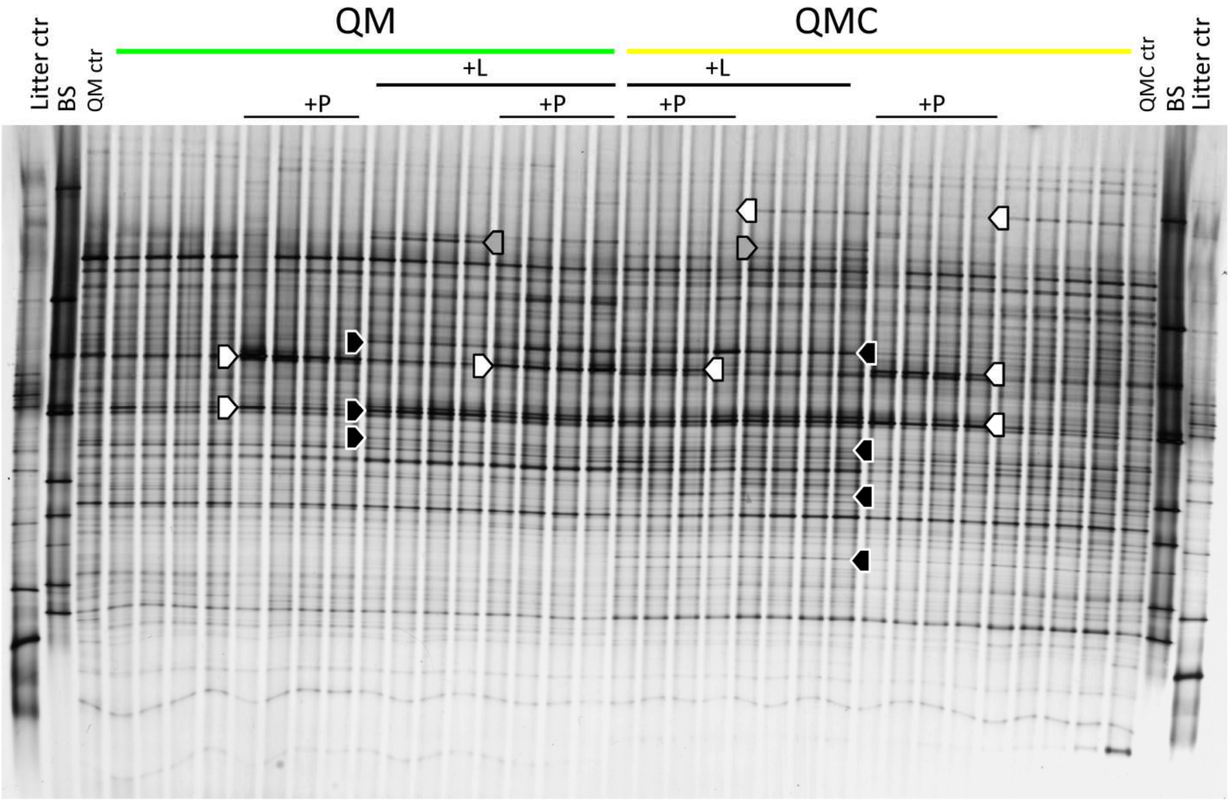

Figure S4. **Response of bacterial communities in QM and QMC soils 63 days after spiking.** DGGE fingerprints of bacterial communities in spiked QM and QMC soils sampled 63 days after spiking (control, phenanthrene (+P), litter (+L), litter and phenanthrene [+L+P]). Arrows mark populations responding to litter (black), phenanthrene (white), litter and phenanthrene (grey). BS-bacterial DGGE standard. QM/QMC ctr-bacterial community in QM or QMC, respectively, before spiking. Litter ctr-control fingerprint of litter used for amendments. Q-quartz, M-montmorillonite, C-charcoal.
