# Supplementary material for: Soil Mineral Composition Matters: Response of Microbial Communities to Phenanthrene and Plant Litter Addition in Long-Term Matured Artificial Soils
Source: PLoS One. 2014 Sep 15;9(9):e106865. doi: 10.1371/journal.pone.0106865 (PMC4164357; doi:10.1371/journal.pone.0106865)
Supplement: Figure S5 — Dendrograms of Alphaproteobacteria and Pseudomonas communities in artificial soils. UPGMA cluster analysis of DGGE fingerprints of taxon-specific 16S rRNA gene amplicons from artificial soils a) QM and QMC b) QI and QIF sampled 21 days after spiking (control, phenanthrene (+P), litter (+L), litter and phenanthrene [+L+P]). Q-quartz, M-montmorillonite, C-charcoal, I-illite, F-ferrihydrite. (PDF) [file pone.0106865.s005.pdf]

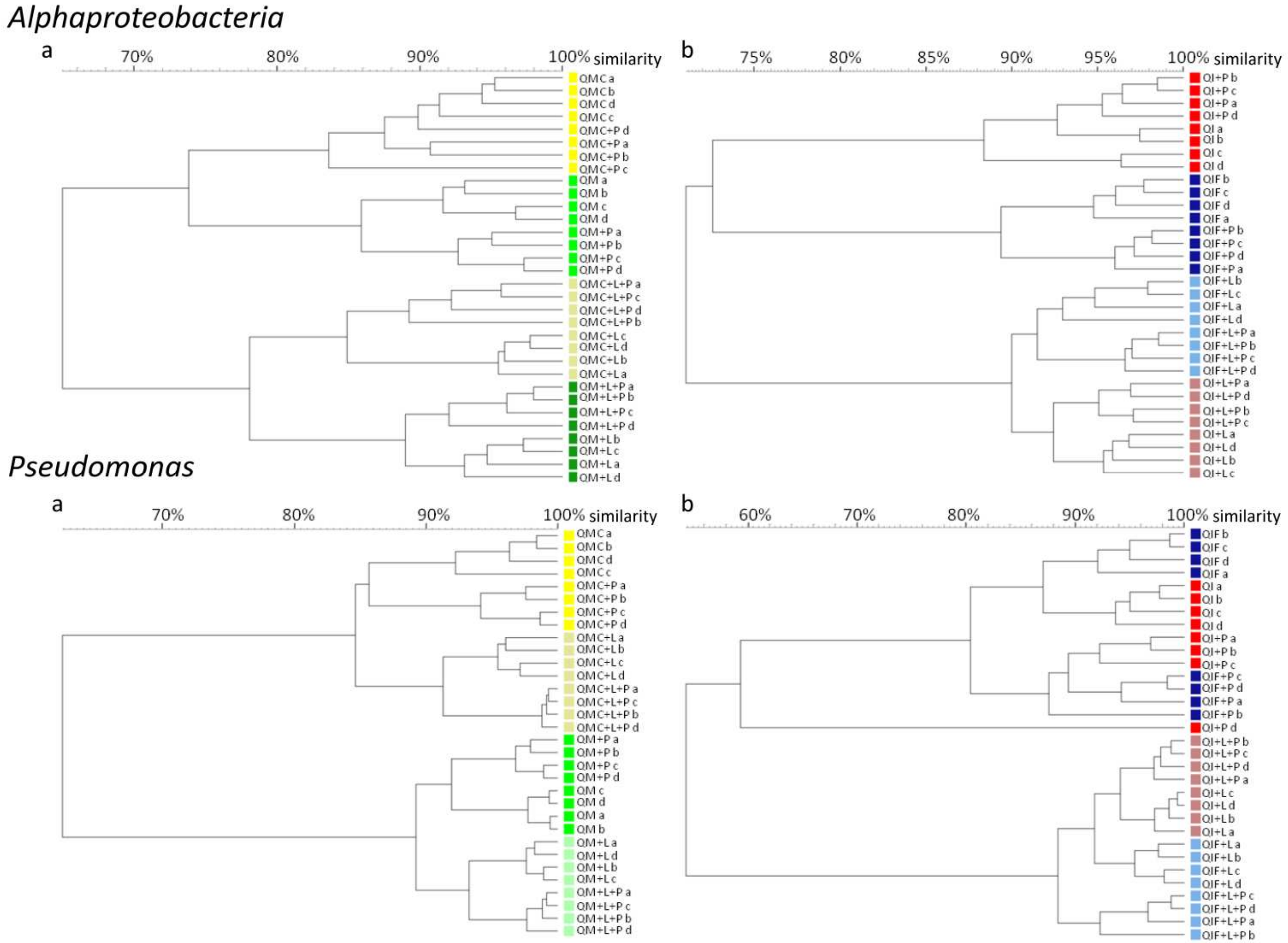

Figure S5. **Dendrograms of *Alphaproteobacteria* and *Pseudomonas* communities in artificial soils.** UPGMA cluster analysis of DGGE fingerprints of taxon-specific 16S rRNA gene amplicons from artificial soils a) QM and QMC b) QI and QIF sampled 21 days after spiking (control, phenanthrene (+P), litter (+L), litter and phenanthrene [+L+P]). Q-quartz, M-montmorillonite, C-charcoal, I-illite, F-ferrihydrite.
