# Supplementary material for: Soil Mineral Composition Matters: Response of Microbial Communities to Phenanthrene and Plant Litter Addition in Long-Term Matured Artificial Soils
Source: PLoS One. 2014 Sep 15;9(9):e106865. doi: 10.1371/journal.pone.0106865 (PMC4164357; doi:10.1371/journal.pone.0106865)
Supplement: Figure S6 — Response of Actinobacteria in QM and QMC soils to spiking. DGGE fingerprints of actinobacterial communities in spiked QM and QMC soils sampled 21 days after spiking (control, phenanthrene (+P), litter (+L), litter and phenanthrene [+L+P]). Arrows mark populations responding to litter (black), phenanthrene (white). BS-bacterial DGGE standard. Ino-Luvisol inoculant added to soil mixtures at the beginning of incubation. Q-quartz, M-montmorillonite, C-charcoal. (PDF) [file pone.0106865.s006.pdf]

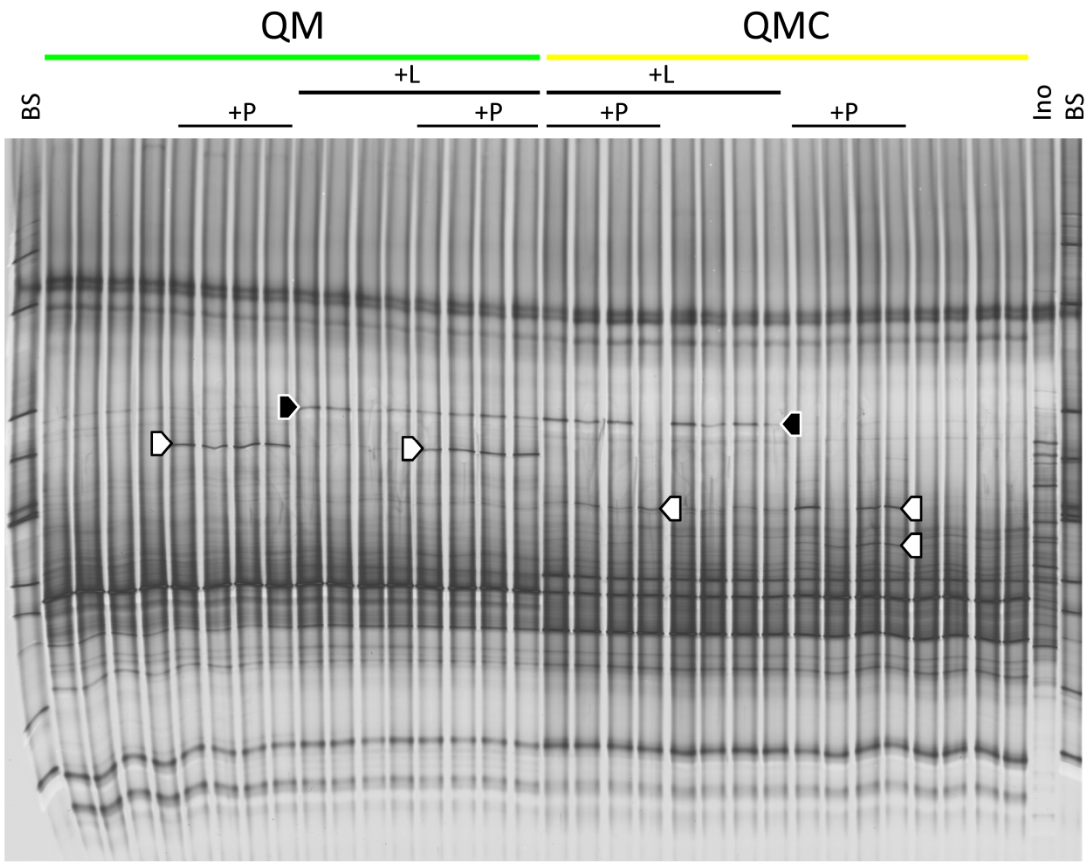

Figure S6. **Response of *Actinobacteria* in QM and QMC soils to spiking**. DGGE fingerprints of actinobacterial communities in spiked QM and QMC soils sampled 21 days after spiking (control, phenanthrene (+P), litter (+L), litter and phenanthrene [+L+P]). Arrows mark populations responding to litter (black), phenanthrene (white). BS-bacterial DGGE standard. Ino-Luvisol inoculant added to soil mixtures at the beginning of incubation. Q-quartz, M-montmorillonite, C-charcoal.
