# Supplementary material for: Soil Mineral Composition Matters: Response of Microbial Communities to Phenanthrene and Plant Litter Addition in Long-Term Matured Artificial Soils
Source: PLoS One. 2014 Sep 15;9(9):e106865. doi: 10.1371/journal.pone.0106865 (PMC4164357; doi:10.1371/journal.pone.0106865)
Supplement: Figure S7 — Response of fungal communities to spiking in QM and QMC soils. DGGE fingerprints of fungal communities in spiked QM and QMC soils sampled 63 days after spiking (control, phenanthrene (+P), litter (+L), litter and phenanthrene [+L+P]). Arrows mark populations responding to litter (black), litter and phenanthrene (grey). FS-fungal DGGE standard. QM/QMC ctr-fungal community in QM or QMC, respectively, before spiking. Q-quartz, M-montmorillonite, C-charcoal. (PDF) [file pone.0106865.s007.pdf]

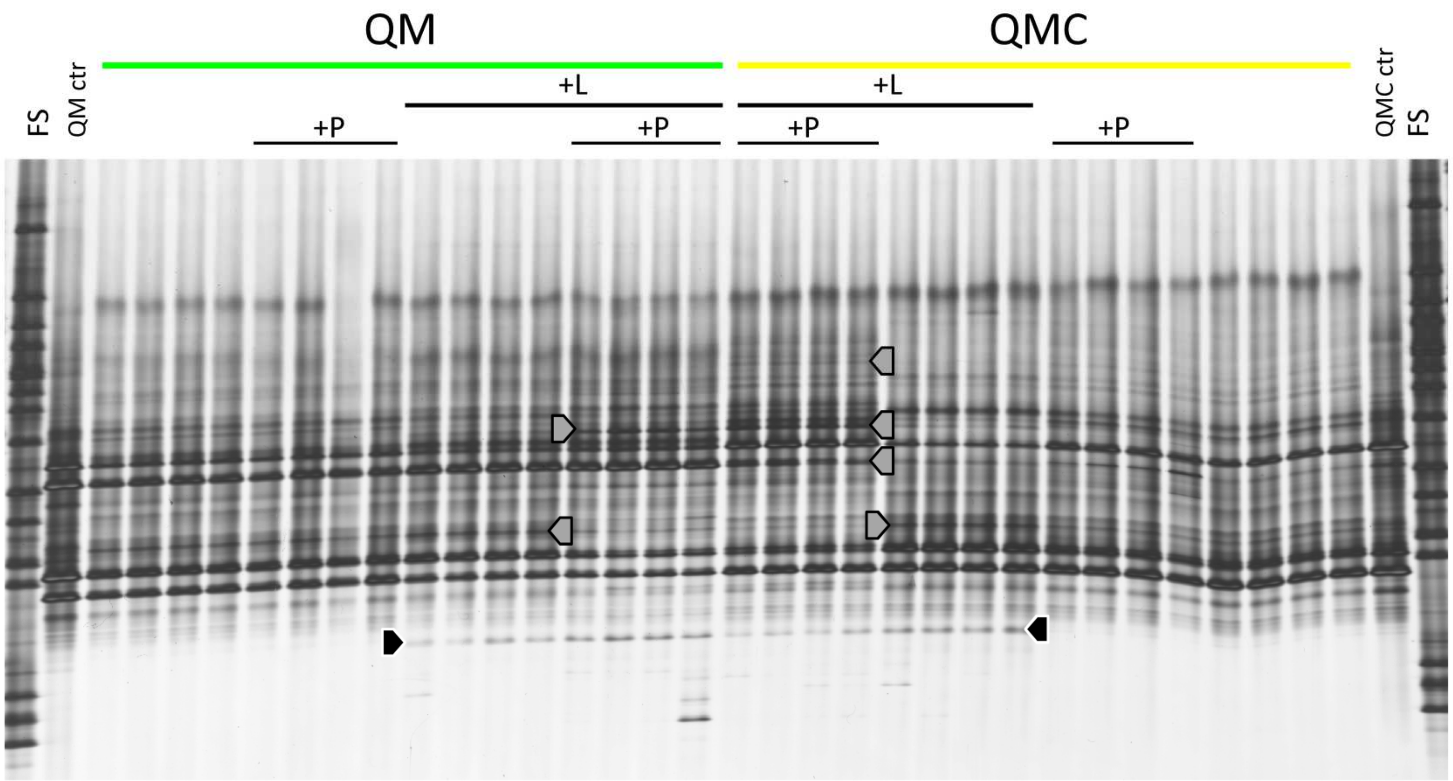

Figure S7. **Response of fungal communities to spiking in QM and QMC soils.** DGGE fingerprints of fungal communities in spiked QM and QMC soils sampled 63 days after spiking (control, phenanthrene (+P), litter (+L), litter and phenanthrene [+L+P]). Arrows mark populations responding to litter (black), litter and phenanthrene (grey). FS-fungal DGGE standard. QM/QMC ctr-fungal community in QM or QMC, respectively, before spiking. Q-quartz, M-montmorillonite, C-charcoal.
