# Supplementary material for: Soil Mineral Composition Matters: Response of Microbial Communities to Phenanthrene and Plant Litter Addition in Long-Term Matured Artificial Soils
Source: PLoS One. 2014 Sep 15;9(9):e106865. doi: 10.1371/journal.pone.0106865 (PMC4164357; doi:10.1371/journal.pone.0106865)
Supplement: Figure S8 — Response of bacterial communities to spiking in the natural soil. DGGE fingerprints of bacterial communities in the spiked natural Luvisol soil (Luv) sampled 63 days after spiking (control, phenanthrene (+P), litter (+L), litter and phenanthrene [+L+P]). Arrows mark populations responding to litter (black), phenanthrene (white). BS-bacterial DGGE standard. Luv ctr-bacterial community in the natural soil before spiking. (PDF) [file pone.0106865.s008.pdf]

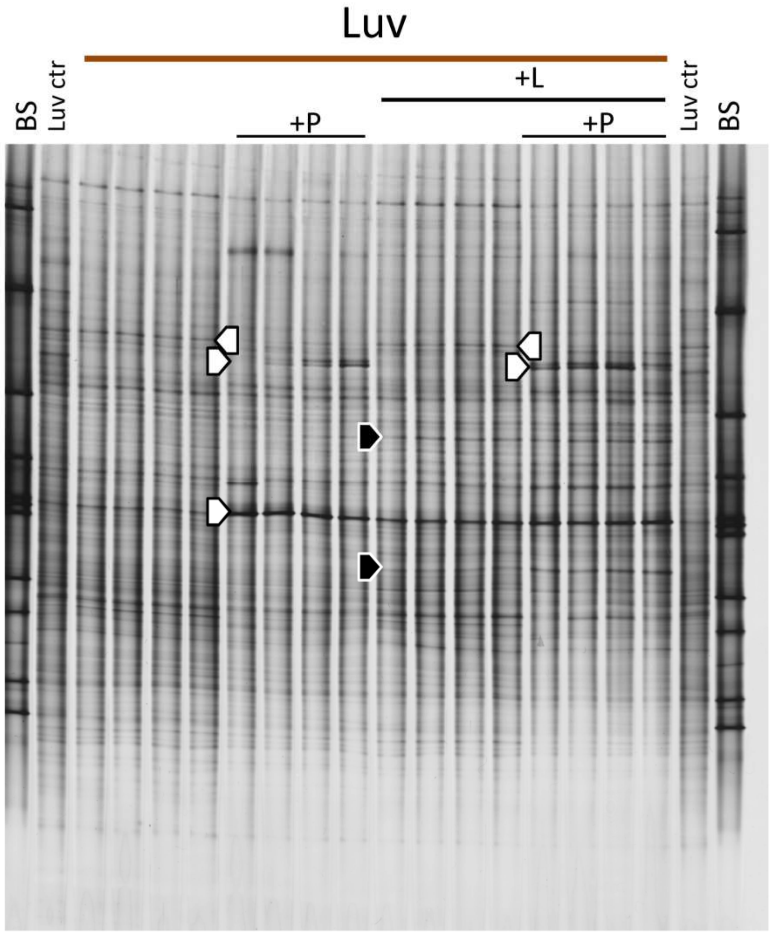

Figure S8. **Response of bacterial communities to spiking in the natural soil.** DGGE fingerprints of bacterial communities in the spiked natural Luvisol soil (Luv) sampled 63 days after spiking (control, phenanthrene (+P), litter (+L), litter and phenanthrene [+L+P]). Arrows mark populations responding to litter (black), phenanthrene (white). BS-bacterial DGGE standard. Luv ctr-bacterial community in the natural soil before spiking.
