# Supplementary material for: Soil Mineral Composition Matters: Response of Microbial Communities to Phenanthrene and Plant Litter Addition in Long-Term Matured Artificial Soils
Source: PLoS One. 2014 Sep 15;9(9):e106865. doi: 10.1371/journal.pone.0106865 (PMC4164357; doi:10.1371/journal.pone.0106865)
Supplement: Table S1 — Primers and probe used in the study. (PDF) [file pone.0106865.s009.pdf]

Table S1: Primers and probe used in the study.

Template		Primer	Sequence 5’ → 3’	Annealing temp [°C]	Reference
**Bacterial 16S rRNA gene fragment**		F984GC	GC clamp[1] AACGCGAAGAACCTTAC	53	[1]
		R1378	CGGTGTGTACAAGGCCCGGGAACG		[2]
**Fungal ITS fragment**	1st PCR	ITS1F	CTTGGTCATTTAGAGGAAGTAA	55	[3]
		ITS4	TCCTCCGCTTATTGATATGC		[4]
	2nd PCR	ITS1F-GC	GC clamp[2] CTTGGTCATTTAGAGGAAGTAA	55	[3]
		ITS2	GCTGCGTTCTTCATCGATGC		[4]
**16S rRNA gene fragment of specific bacterial groups**	*Alphaproteobacteria*	F203α*	CCGCATACGCCCTACGGGGGAAAGATTTAT	56	[5]
	Betaproteobacteria	F948β*	CGCACAAGCGGTGGATGA	64	[5]
	Actinobacteria	F243HGC*	GGATGAGCCCGCGGCCTA	63	[2]
		*Reverse primer R1494	CTACGGYTACCTTGTTACGAC		[6]
	Pseudomonas	F311Ps	CTGGTCTGAGAGGATGATCAGT	63	[7]
		R1459Ps	AATCACTCCGTGGTAACCGT		
**Quantitative real-time PCR**	bacterial 16S rRNA gene fragment	Bact1369F	CGGTGAATACGTTCYCGG	56	[8]
		Prok1492R	GGWTACCTTGTTACGACTT		
		TM1389F (5’-FAM, 3’-TAMRA)	CTTGTACACACCGCCCGTC		
	fungal ITS fragment	ITS1	TCC GTA GGT GAA CCT GCG G	50	[4]
		ITS4	TCCTCCGCTTATTGATATGC		

1 bacterial GC clamp: CGC-CCG-GGG-CGC-GCC-CCG-GGC-GGG-GCG-GGG-GCA-CGG-GGG [1]

2 fungal GC clamp: CGC-CCG-CCG-CGC-GCG-GCG-GGC-GGG-GCG-GGG-GCA-CGG-GGG-G [9]

References

1. Nübel U, Engelen B, Felske A, Snaidr J, Wieshuber A, et al. (1996) Sequence heterogeneities of genes encoding 16S rRNAs in Paenibacillus polymyxa detected by temperature gradient gel electrophoresis. J Bacteriol 178: 5636-5643.

2. Heuer H, Krsek M, Baker P, Smalla K, Wellington EM (1997) Analysis of actinomycete communities by specific amplification of genes encoding 16S rRNA and gel-electrophoretic separation in denaturing gradients. Appl Environ Microbiol 63: 3233-3241.

3. Gardes M, Bruns TD (1993) ITS primers with enhanced specificity for basidiomycetes-application to the identification of mycorrhizae and rusts. Mol Ecol 2: 113-118.

4. White TJ, Bruns T, Lee S, Taylor J (1990) Amplification and direct sequencing of fungal ribosomal RNA genes for phylogenetics. In: Innis M, Gelfand D, Sninsky J, White T, editors. PCR Protocols: A guide to Methods and Applications. Orlando, Florida: Academic Press pp. 315-322.

5. Gomes NCM, Heuer H, Schönfeld J, Costa R, Mendonça-Hagler L, et al. (2001) Bacterial diversity of the rhizosphere of maize (*Zea mays*) grown in tropical soil studied by temperature gradient gel electrophoresis. Plant Soil 232: 167-180.

6. Weisburg WG, Barns SM, Pelletier DA, Lane DJ (1991) 16S ribosomal DNA amplification for phylogenetic study. J Bacteriol 173: 697-703.

7. Milling A, Smalla K, Maidl FX, Schloter M, Munch JC (2004) Effects of transgenic potatoes with an altered starch composition on the diversity of soil and rhizosphere bacteria and fungi. Plant Soil 266: 23-39.

8. Suzuki MT, Taylor LT, DeLong EF (2000) Quantitative analysis of small-subunit rRNA genes in mixed microbial populations via 5'-nuclease assays. Appl Environ Microbiol 66: 4605-4614.

9. Muyzer G, de Waal EC, Uitterlinden AG (1993) Profiling of complex microbial populations by denaturing gradient gel electrophoresis analysis of polymerase chain reaction-amplified genes coding for 16S rRNA. Appl Environ Microbiol 59: 695-700.
