# Supplementary material for: Soil Mineral Composition Matters: Response of Microbial Communities to Phenanthrene and Plant Litter Addition in Long-Term Matured Artificial Soils
Source: PLoS One. 2014 Sep 15;9(9):e106865. doi: 10.1371/journal.pone.0106865 (PMC4164357; doi:10.1371/journal.pone.0106865)
Supplement: Table S2 — Percent difference (d-values) between bacterial communities of different artificial soils per treatment (control, phenanthrene (+P), litter (+L), litter and phenanthrene [+L+P]) 21 days after spiking. D-values were calculated based on pairwise Pearson similarity coefficients and were significant for all comparisons (p<0.05). Q-quartz, M-montmorillonite, C-charcoal, I-illite, F-ferrihydrite. (PDF) [file pone.0106865.s010.pdf]

Table S2: Percent difference (d-values) between bacterial communities of different artificial soils per treatment (control, phenanthrene (+P), litter (+L), litter and phenanthrene [+L+P]) 21 days after spiking. D-values were calculated based on pairwise Pearson similarity coefficients and were significant for all comparisons ($p<0.05$). Q-quartz, M-montmorillonite, C-charcoal, I-illite, F-ferrihydrite.

% difference	control	+P	+L	+L+P
QM vs. QMC	13.25	33.16	30.52	26.75
QM vs. QI	40.62	55.1	38.86	34.65
QM vs. QIF	38.53	70.05	46.43	45.28
QMC vs. QI	29.51	33.34	25.08	23.83
QMC vs. QIF	24.66	52.73	40.03	32.18
QI vs. QIF	16.54	29.44	13.19	15.69
